# Supplementary material for: Inflammatory-miR-301a circuitry drives mTOR and Stat3-dependent PSC activation in chronic pancreatitis and PanIN
Source: Mol Ther Nucleic Acids. 2022 Jan 19;27:970–82. doi: 10.1016/j.omtn.2022.01.011 (PMC8829454; doi:10.1016/j.omtn.2022.01.011)
Supplement: Document S1. Figures S1–S6 and Table S1 [file mmc1.pdf]

OMTN, Volume *27*

Supplemental information

Inflammatory-miR-301a circuitry drives mTOR and Stat3-dependent PSC activation in chronic pancreatitis and PanIN

Fugui Li, Miaomiao Wang, Xun Li, Yihao Long, Kaizhao Chen, Xinjie Wang, Mingtian Zhong, Weimin Cheng, Xuemei Tian, Ping Wang, Mingfang Ji, and Xiaodong Ma

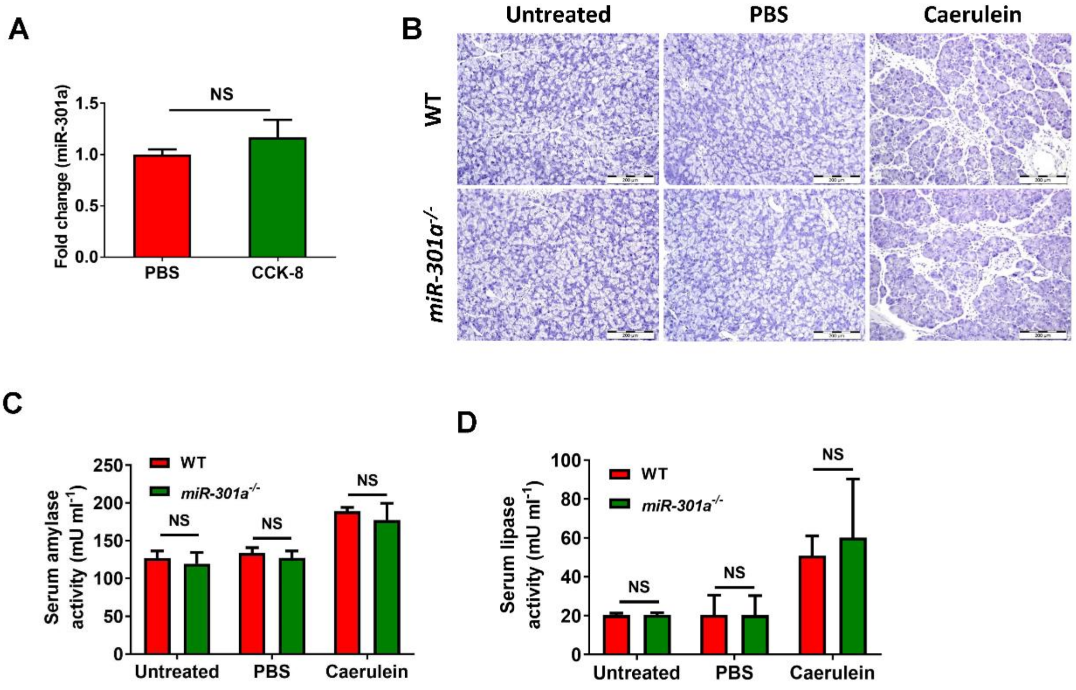

Figure S1. Response of WT and *miR-301a*$^{-/-}$ mice to caerulein-induced acute pancreatitis.

(A) Expression of miR-301a in acinar cells treated with supramaximal CCK-8. Acinar cells were isolated from WT and *miR-301a*$^{-/-}$ mice, and incubated for 8h with CCK-8. Total RNA was extracted from acinar cells and miR-301a expression was quantified by qPCR. **(B)** Representative histological sections of mouse pancreata stained with haematoxylin from caerulein-treated WT and *miR-301a*$^{-/-}$ mice (n=6 per group). Scale bars, 200μm. **(C)** Serum amylase activity in WT and *miR-301a*$^{-/-}$ mice (n=6 per group) 6h after the last caerulein injection. **(D)** Serum lipase activity in WT and *miR-301a*$^{-/-}$ mice (n=6 per group) 6h after the last caerulein injection. Values are mean±s.d. NS means no significant difference between the indicated groups.

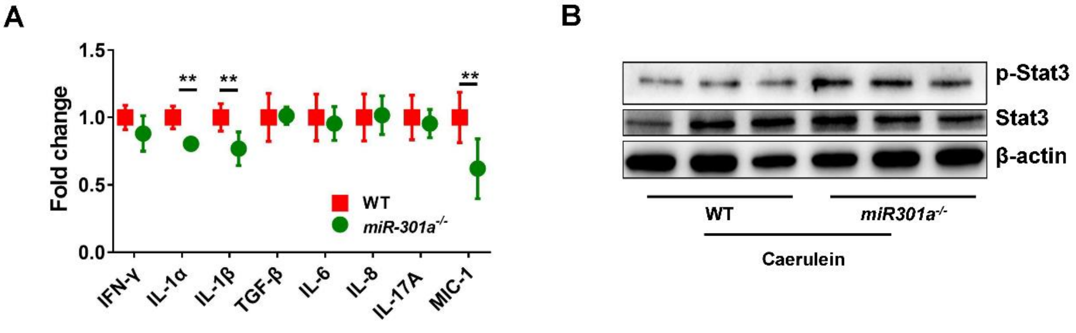

Figure S2. Stat3 activation and cytokines expression in pancreata from *miR-301a$^{-/-}$* mice compared to WT mice with chronic pancreatitis.

(A) Cytokine gene expression in pancreatic tissues of WT and *miR-301a$^{-/-}$* mice as determined by qPCR (n=3 per group). **(B)** Western blot analysis of pancreatic tissue lysates for Stat3 activation (n=3 per group). $^{**}P<0.01$ indicates a significant difference between the indicated groups.

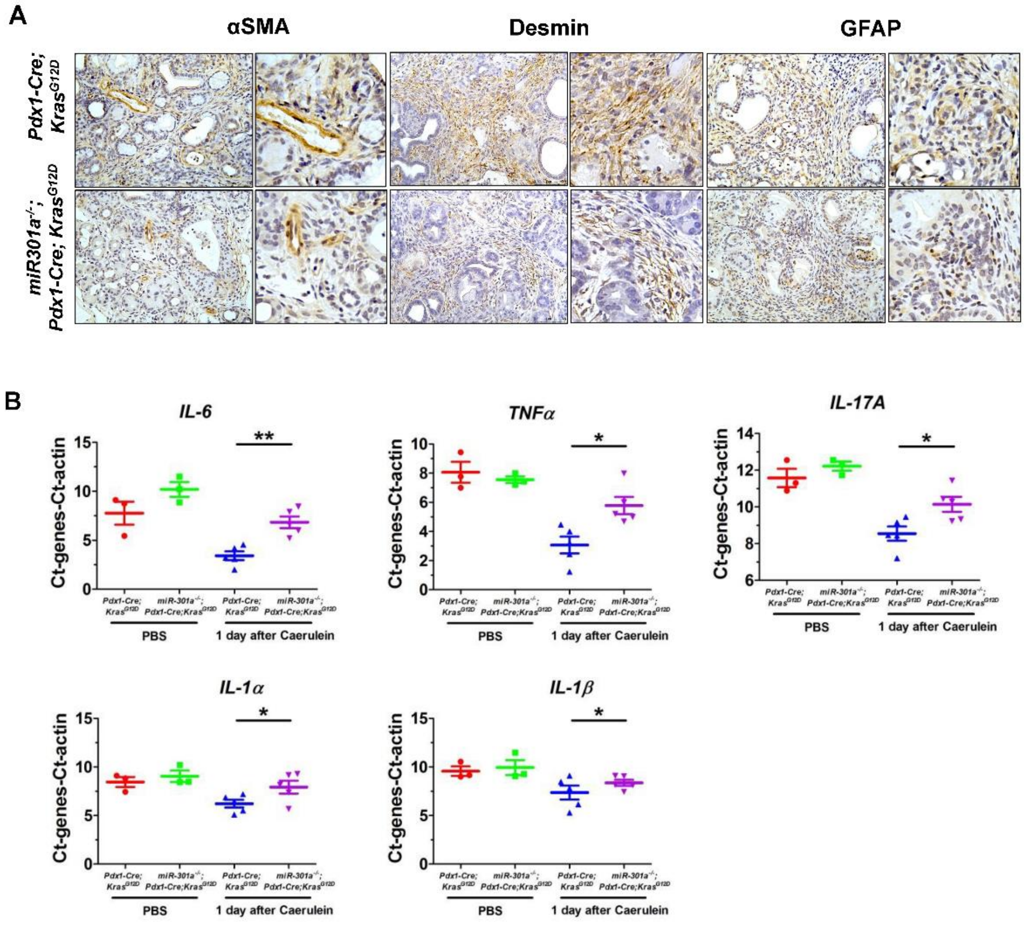

Figure S3. Characteristic markers and cytokines expression in PanIN.

(A) Immunohistochemical staining of αSMA**,** Desmin and GFAP showed activation of PSCs and increased fibrogenesis pancreata from *Pdx1-Cre;Kras*^{G12D} (n=8) and *miR-301a*$^{-/-}$*;Pdx1-Cre;Kras*^{G12D} (n=8) littermate mice at 18 weeks of age. Scale bars, 75μm. Values are means ± s.d. **(B)** Cytokine gene expression in pancreatic tissues from 9-week-old *Pdx1-Cre;Kras*^{G12D} and *miR-301a*$^{-/-}$*;Pdx1-Cre;Kras*^{G12D} mice 7 days after PBS (n=3) or caerulein treatment (n=5). ***P*<0.01 or **P*<0.05 indicates a significant difference between the indicated groups.

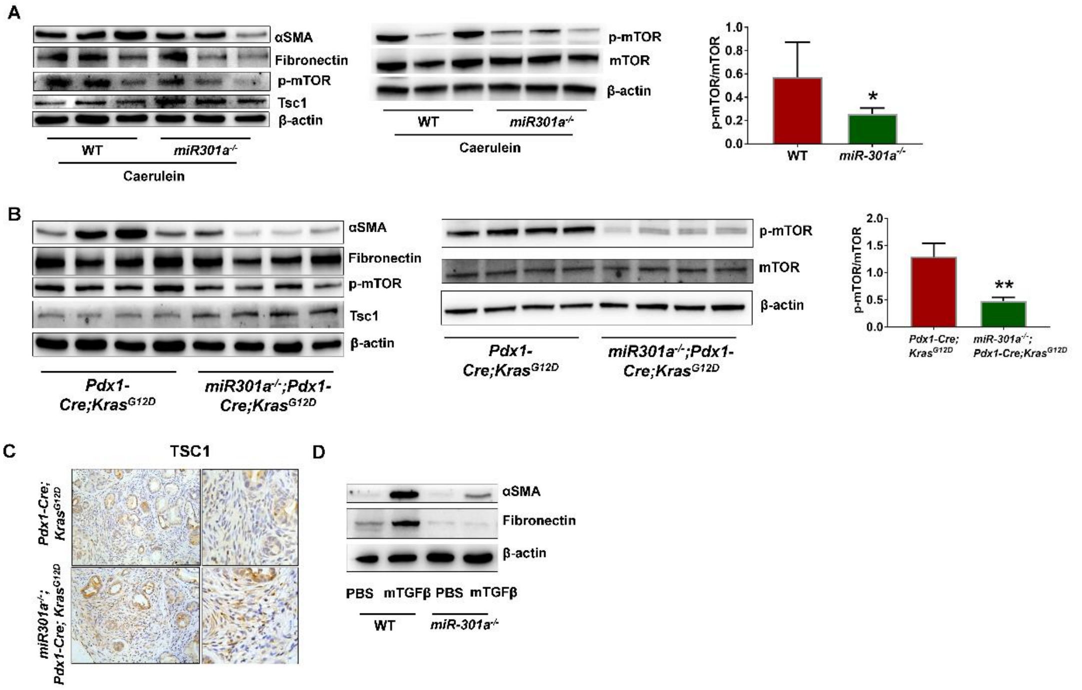

Figure S4. miR-301a/Tsc1 in chronic pancreatitis and spontaneous PanIN.

(A) Western blot shows the expression of αSMA, Fibronectin, p-mTOR， mTOR and Tsc1 in pancreatic tissues from caerulein-treated WT (n=3) and *miR-301a*$^{-/-}$ (n=3) mice. **(B)** Western blot shows the expression of αSMA, Fibronectin, p-mTOR， mTOR and Tsc1 in pancreatic tissues from *Pdx1-Cre;Kras*^{G12D} (n=4) and *miR-301a*$^{-/-}$*;Pdx1-Cre;Kras*^{G12D} (n=4) littermate mice at 18 weeks of age. **(C)** Immunohistochemical staining of Tsc1 in pancreata from 9-week-old *Pdx1-Cre;Kras*^{G12D} (n=8) and *miR-301a*$^{-/-}$*;Pdx1-Cre;Kras*^{G12D} (n=8) mice 7 days after caerulein treatment. The scale bars represent 75μm. **(D)** MEFs isolated from WT and *miR-301a*$^{-/-}$ mice were treated with TGF-β (10 ng/mL) for 24h. The expression of αSMA and fibronectin were measured by western blot analysis. **P<0.01 or *P<0.05indicate a significant difference between normal tissue and tumor tissue.

A

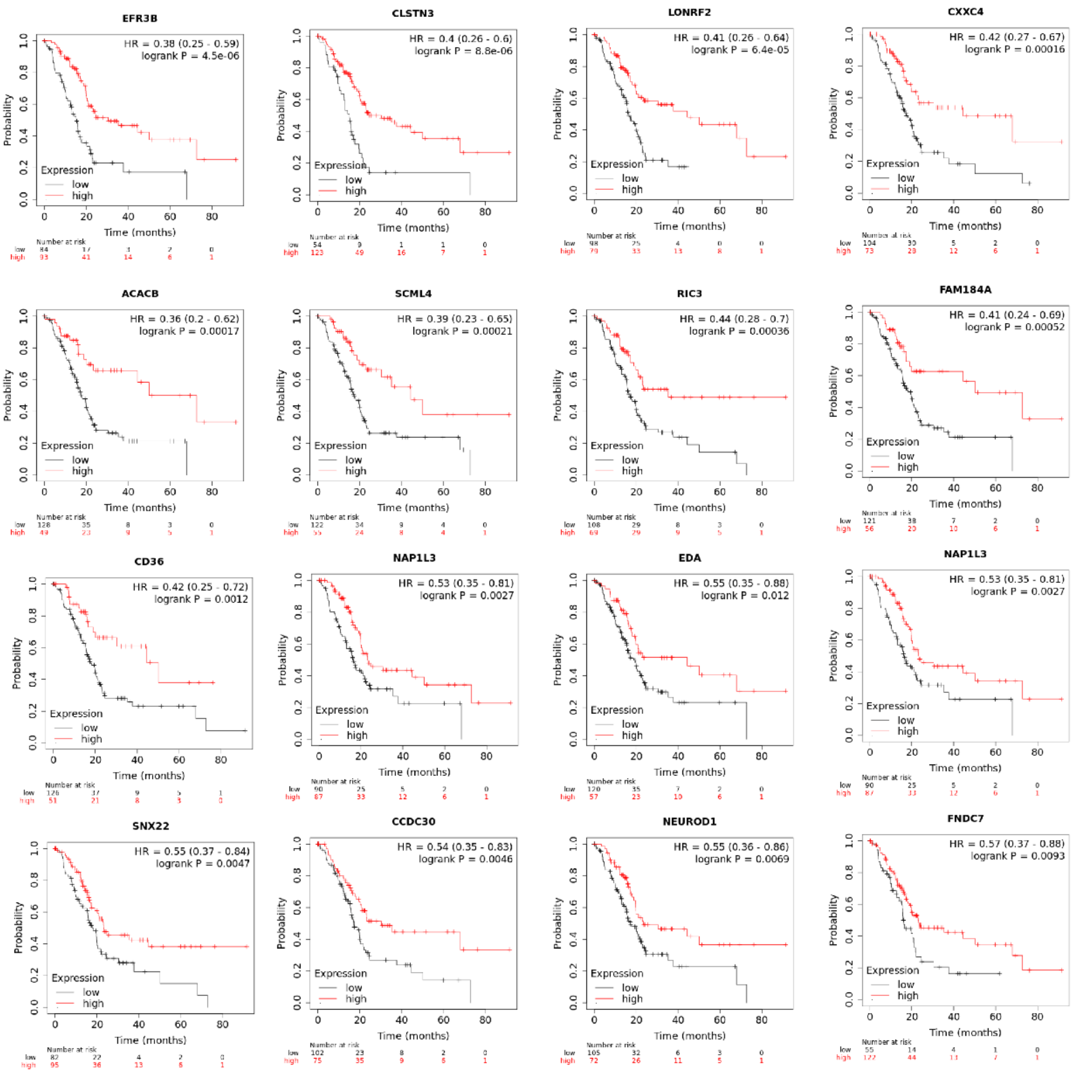

Figure S5. **(A)** Survival plots of human pancreatic patients (TCGA, PAAD) for 16 genes.

A

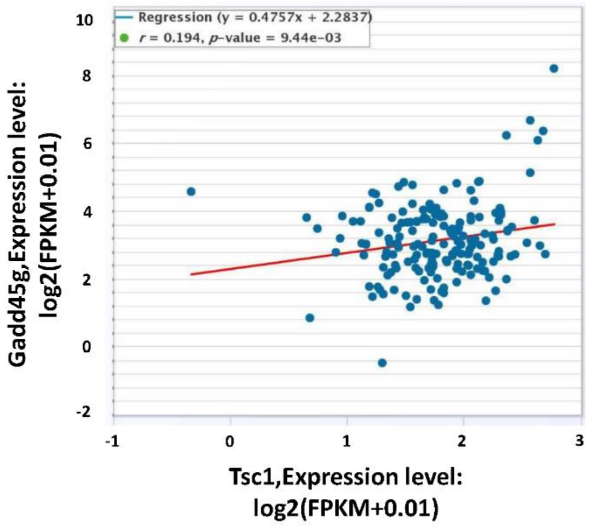

Figure S6. (A) Correlation analyses between Gadd45g and Tsc1 in pancreatic cancer patients in the TCGA database.

Table S1 The primers used for qPCR assay.

Genes	Forward	Reverse
NeuroD1	5'-AAGCCACGGATCAATCTTCTC-3'	5'-CGTGAAAGATGGCATTAAGCTG-3'
Acacb	5'-GGTAGTGGCTTTGAAGGAACTGTC-3'	5'-GATATCGTTGTTCTGGAAGCTCTCG-3'
Chst2	5'-AAGCCTACAGGTGGTGCGAA-3'	5'-CAGGACTGTTAACCCGCTCA-3'
Cd36	5'-GAGCCATCTTTGAGCCTTCA-3'	5'-TCAGATCCGAACACAGCGTA-3'
Ric3	5'-GGAAACGCCCACAGGAAGATT-3'	5'-ACCATTAGGTCCAACTCTGTTGA-3'
Ccdc30	5'-AAGGCTTGTTCAGACTAGCTCT-3'	5'-TGTAGGCTGCTGTTTGGATCT-3'
Fam184a	5'-AGCTGGCAACATCACTATTACG-3'	5'-GCTGGGCGATCTTCTTGCT-3'
Fndc7	5'-ATCACCGTAGAATGGACTACCG-3'	5'-CCTGGGGAATTAGCCACTGT-3'
Clstn3	5'-GGACAAGGCAACGGGTGAA-3'	5'-GCCACAGTCATAAGCCTGAATG-3'
Nap1l3	5'-GGGGATGAATCTGACAGCAATAG-3'	5'-TTCGGTTCAAATGACTGCCAT-3'
Snx22	5'-TACACAAGCGGATCAAGAAACG-3'	5'-TGGATGTAGGTCTCTAATCCCTG-3'
Scml4	5'-GTGCAGATGGCCGTCCATAA-3'	5'-AGCACTGGAGACTTGATCTTGT-3'
Gadd45g	5'-GGGAAAGCACTGCACGAACT-3'	5'-AGCACGCAAAAGGTCACATTG-3'
Lonrf2	5'-CAACTCGGCGAAGAAAGAAACA-3'	5'-AGGATACTGGGGATTCGTCTG-3'
Cxxc4	5'-CTGCCCGCAGAATCATTCCT-3'	5'-CAGACGCCACAGTTGATGAG-3'
Efr3b	5'-CGCCTGAAAAACTGGATCGAA-3'	5'-GGCAATACACACGTACCCATATC-3'
Eda	5'-GTGGACGGCACCTACTTCATC-3'	5’-CACCATCTTCACGGCGATTT-3'
IFN-γ	5'-ATGAACGCTACACACTGCATC-3'	5'-CCATCCTTTTGCCAGTTCCTC-3'
IL-1α	5'-CTCTAGAGCACCATGCTACAGAC-3'	5'-TGGAATCCAGGGGAAACACTG-3'
IL-1β	5'-CTCCATGAGCTTTGTACAAGG-3'	5'-TGCTGATGTACCAGTTGGGG-3'
Il-6	5'-CCTCTGGTCTTCTGGAGTACC-3'	5'-ACTCCTTCTGTGACTCCAGC-3'
TGF-β	5'-GCTACCATGCCAACTTCTGT-3'	5'-CGTAGTAGACGATGGGCAGT-3'
IL-17A	5'-TCCCCTCTGTGATCTGGGAAG-3'	5'-AGCATCTTCTCGACCCTGAA-3'
MIC-1	5'-CTGGCAATGCCTGAACAACG-3'	5'-GGTCGGGACTTGGTTCTGAG-3'
IL-8	5'-CAGCTGCCTTAACCCCATCA-3'	5'-CTTGAGAAGTCCATGGCGAAA-3'
