# Supplementary material for: Inflammatory-miR-301a circuitry drives mTOR and Stat3-dependent PSC activation in chronic pancreatitis and PanIN
Source: Mol Ther Nucleic Acids. 2022 Jan 19;27:970–82. doi: 10.1016/j.omtn.2022.01.011 (PMC8829454; doi:10.1016/j.omtn.2022.01.011)
Supplement: Document S2. Article plus supplemental information [file mmc2.pdf]

Molecular Therapy
Nucleic Acids
Original Article

Inflammatory-miR-301a circuitry drives mTOR and Stat3-dependent PSC activation in chronic pancreatitis and PanIN

Fugui Li,[1,4] Miaomiao Wang,[2,4] Xun Li,[2,4] Yihao Long,[2] Kaizhao Chen,[2] Xinjie Wang,[2] Mingtian Zhong,[2] Weimin Cheng,[1] Xuemei Tian,[2] Ping Wang,[3] Mingfang Ji,[1] and Xiaodong Ma[2]

[1]Cancer Research Institute of Zhongshan City, Zhongshan City People's Hospital, 528403 Zhongshan, China; [2]Key Laboratory of Brain, Cognition and Education Sciences, Ministry of Education, Guangdong Key Laboratory of Mental Health and Cognitive Science, Center for Studies of Psychological Application, Institute for Brain Research and Rehabilitation, South China Normal University, 510631 Guangzhou, China; [3]Department of Hepatobiliary Surgery, The First Affiliated Hospital of Guangzhou Medical University, Guangzhou, 510120 Guangdong Province, China

Activated pancreatic stellate cells (PSCs) are the main cells involved in chronic pancreatitis and pancreatic intraepithelial neoplasia lesion (PanIN). Fine-tuning the precise molecular targets in PSC activation might help the development of PSC-specific therapeutic strategies to tackle progression of pancreatic cancer-related fibrosis. miR-301a is a pro-inflammatory micro-RNA known to be activated by multiple inflammatory factors in the tumor stroma. Here, we show that miR-301a is highly expressed in activated PSCs in mice, sustained tissue fibrosis in caerulein-induced chronic pancreatitis, and accelerated PanIN formation. Genetic ablation of miR-301a reduced pancreatic fibrosis in mouse models with chronic pancreatitis and PanIN. Cell proliferation and activation of PSCs was inhibited by downregulation of miR-301a via two of its targets, Tsc1 and Gadd45g. Moreover, aberrant PSC expression of miR-301a and Gadd45g restricted the interplay between PSCs and pancreatic cancer cells in tumorigenesis. Our findings suggest that miR-301a activates two major cell proliferation pathways, Tsc1/mTOR and Gadd45g/Stat3, *in vivo*, to facilitate development of inflammatory-induced PanIN and maintenance of PSC activation and desmoplasia in pancreatic cancer.

INTRODUCTION

Recent advances in our understanding of the fundamental stromal biology in chronic pancreatitis and pancreatic cancer suggest a key role of pancreatic stellate cells (PSCs) in the pathobiology of the major disorders of the exocrine pancreas, including chronic pancreatitis and pancreatic cancer.[1] PSCs are functional myofibroblasts present in the surrounding area of acinar, vascular, and ductal regions.[2,3] Under normal physiological conditions, PSCs are found in a quiescent state throughout the pancreas and exhibit a cytoplasm rich in vitamin A lipid droplets, which is characterized by the expression of glial filament acidic protein (GFAP) and desmin.[4,5] However, upon activation by damage or by growth factor signaling, such as chronic pancreatitis and pancreatic intraepithelial neoplasia (PanIN), PSCs can be transformed into an activated state and participate in the interstitial remodeling of the pancreas.[6,7] Activated PSCs accumulate in the extracellular matrix (ECM) and increase cytokine/chemokine production, which promote aggressive phenotypes and therapy resistance in pancreatic inflammation and cancer.[8,9] For example, supernatant derived from activated PSCs was shown to increase proliferation and migration of cancer cells via SDF-1/CXCR4.[10] Coculture of PSCs with pancreatic cancer cells can also promote cancer proliferation, migration, and invasion via Gal1-driven pathways.[11] Therefore, an in-depth understanding of the precise molecular mechanism underlying PSC activation and their role in pathology might help find new promising treatment strategies for patients with chronic pancreatitis and cancer.

PSC activation often occurs very early during pancreatic injury or pancreatic ductal carcinoma tumorigenesis. Numerous cytokines, growth factors, and intracellular signaling molecules, such as non-coding RNAs, are released during pancreatitis to regulate PSC activation; however, accumulating evidence points to a major role for interleukin-6 (IL-6) and transforming growth factor β (TGF-β) in modulating the persistent activation and fibrotic phenotype of PSCs *in vitro* and *in vivo*. Upregulation of TGF-β in pancreatitis promotes PSC activation in autocrine loops, which leads to ECM synthesis. TGF-β-induced activation of mammalian target of rapamycin (mTOR) activity has also been implicated in multiple fibrotic conditions, including idiopathic pulmonary fibrosis,[12] chronic pancreatitis, and cancer-associated

Received 13 June 2021; accepted 17 January 2022;
https://doi.org/10.1016/j.omtn.2022.01.011.

[4]These authors contributed equally

Correspondence: Ping Wang, Department of Hepatobiliary Surgery, The First Affiliated Hospital of Guangzhou Medical University, Guangzhou, 510120 Guangdong Province, China.
E-mail: Wangping1219@126.com
Correspondence: Mingfang Ji, Cancer Research Institute of Zhongshan City, Zhongshan City People's Hospital, 528403 Zhongshan, China.
E-mail: jmftbh@sina.com
Correspondence: Xiaodong Ma, Institute for Brain Research and Rehabilitation, South China Normal University, Guangzhou 510006, China.
E-mail: sciencema@hotmail.com

 © 2022 The Authors.
This is an open access article under the CC BY license (http://creativecommons.org/licenses/by/4.0/).

fibrosis.[13,14] TGF-β-overexpressing transgenic mice exhibit morphological features of chronic pancreatitis,[15] and depletion of TGF-β expression was shown to hamper the active PSC phenotype, suggesting a role for TGF-β in pancreatic fibrosis through PSC activation. IL-6/Stat3 have also been implicated in pancreatic ductal adenocarcinoma (PDAC), and paracrine IL-6/Stat3 signaling was shown to regulate the effects of PSCs in matricellular fibrosis and tumor progression.[16] Epithelial Stat3 ablation attenuated fibrosis and tumor progression in Smad4 mutant pancreatic tumors in mice, and several studies demonstrated that blockage of the IL-6/Stat3 axis suppresses Kras-induced pancreatic adenocarcinoma.[17,18] Recently, supernatant derived from activated PSC-conditioned medium was shown to stimulate Stat3 activation in pancreatic cancer cells, leading to the accumulation of ECM proteins and exacerbation of tumorigenesis and drug resistance.[11,19] These findings suggest that inhibiting paracrine communication between PSCs and pancreatic cancer cells could be a promising therapeutic target for fibrotic pancreatitis and PDAC.

So far, over 1,000 human microRNAs (miRNAs) have been reported, and miRNAs are predicted to target up to 92% of all human genes.[20] Several miRNAs have been shown to modulate mTOR or Stat3 activation,[21] including miR-7[22] and miR-503,[23] which negatively regulate mTOR signaling to inhibit adult β-cell proliferation. miR-99 family members are involved in dermal wound healing by modulating Akt/mTOR signaling pathways.[24] Interestingly, plant miRNAs also have the ability to modulate human mTOR signaling.[25] Despite extensive investigation of the role of miRNAs in mTOR or Stat3 activation, there is limited research on the role of miRNAs in the pathology of cancer-associated fibrosis using animal models. miR-301a was found to be overexpressed in inflammation-related diseases, in particular inflammation-associated carcinogenesis. As a pro-inflammatory miRNA, miR-301a was shown to facilitate tumorigenesis by activating nuclear factor κB (NF-κB) and Stat3 *in vivo*.[26] miR-301 inhibition reduced the activity of NF-κB and Stat3 in both tumor and immune cells. Our previous study demonstrated that ablation of miR-301a in mice protected against bleomycin-induced lung fibrosis through the Tsc1/mTOR axis signaling pathway.[12] However, whether miR-301a has an anti-fibrogenic function in pancreatitis and pancreatic cancer is unknown.

Growth arrest and DNA-damage-induced 45 gamma (Gadd45g) is epigenetically silenced in many tumors. In hepatocellular carcinoma, Gadd45g elicited cellular senescence and suppressed tumor growth *in vivo*.[27] Upregulation of Gadd45g impaired homologous recombination DNA repair and dramatically induced tumor cell apoptosis without affecting normal cells.[28] In addition, Gadd45g is known to be induced by several cytokines and mediated anti-tumor immune responses.[29–31] Recently, Gadd45g was shown to impair Jak/Stat3 activation in both malignant solid tumor and lymphoid cells, suggesting its critical involvement in tumorigenesis.[27,32]

In the present study, we report that miR-301a deficiency inhibits PSC activation and protects mice from chronic pancreatitis and pancreatitis-associated PanIN. We show that Tsc1/mTOR is targeted by miR-301a in fibrotic pancreatic tissues, and identify Gadd45g as a new target of miR-301a. We suggest that Gadd45g restrains the interaction between myofibroblast PSCs and pancreatic cancer cells by weakening Stat3 activation during PanIN formation. Our findings demonstrate the complex role of miR-301a in chronic inflammation-driven PanIN and PDAC and provide insights that might be helpful in devising new treatments for patients with chronic pancreatitis and cancer.

RESULTS

Overexpression of miR-301a during pancreatitis and pancreatic intraepithelial neoplasia

To investigate the role of miR-301a in acute and chronic pancreatitis, we first measured miR-301a expression in wild-type (WT) mice treated with different doses of caerulein. We found that miR-301a expression was significantly increased in pancreas from mice treated with caerulein. Interestingly, compared with PBS-treated controls, the induction of miR-301a in pancreas revealed a 2.37-fold upregulation under acute pancreatitis conditions and a 7.26-fold change under chronic inflammation conditions (Figures 1A and 1B). Of note, miR-301a expression was no different in isolated acinar cells between PBS and cholecystokinin-8 treatment, which recapitulate necrosis of acute pancreatitis *in vitro* (Figure S1A). Pancreatic intraepithelial lesions are often accompanied by inflammation and expression of multiple inflammatory factors that accelerate the malignant process. Next, we measured the miR-301a expression level in PanIN by using *Pdx1-Cre*;*Kras*^{G12D} mice. These animals develop multifocal pancreatic inflamed lesions and extensive fibrosis. miR-301a was highly expressed both in spontaneous and pancreatitis-accelerated PanIN lesions (Figures 1C and 1D). Moreover, expression of miR-301a and two inflammatory factors, IL-6 and TGF-β, were also found to be significantly higher in serum of patients with chronic pancreatitis compared with healthy donors (Figures 1E–1G). Accordingly, the expression level of miR-301a was positively correlated with IL-6 and TGF-β concentration in patient serum, suggesting the key role of miR-301a in pancreatic fibrosis and malignant process (Figures 1H and 1I).

Response of WT and *miR-301a*$^{-/-}$ mice to caerulein-induced pancreatitis

To investigate the role of miR-301a in acute and chronic pancreatitis, we examined pancreata in both WT and *miR-301a*$^{-/-}$ mice treated with caerulein. We first evaluated changes in pancreatic histology at 1 day after the last caerulein injection by using the acute pancreatitis protocol. The severity of acute pancreatitis in WT mice treated with caerulein was similar to that found in *miR-301a*$^{-/-}$ mice (Figure S1B). Both serum amylase and lipase activity were not significantly different in *miR-301a*$^{-/-}$ mice and WT mice after the final dose of caerulein (Figures S1C and S1D).

Next, we evaluated the level of pancreatic inflammation and fibrosis, which are typical pathological features in chronic pancreatitis, in WT and *miR-301a*$^{-/-}$ mice with continuous caerulein injection for 3 weeks (Figure 2A). Our results show that pancreatic fibrosis is significantly decreased in *miR-301a*$^{-/-}$ mice compared

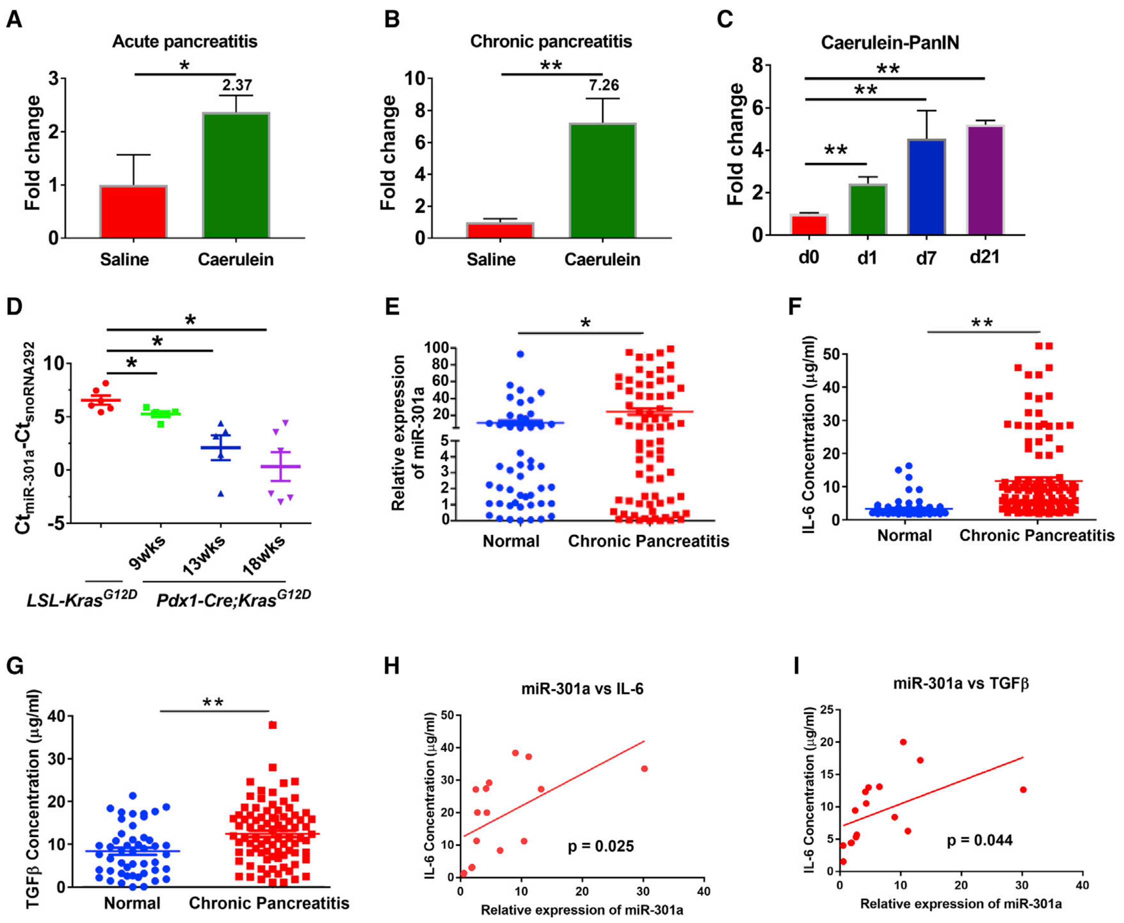

Figure 1. miR-301a expression during caerulein-induced pancreatitis and PanIN formation

(A) Expression of miR-301a during caerulein-induced acute pancreatitis in WT mice (n = 5 per group). Pancreatic tissues were harvested at day 1 after the last caerulein or saline (control) injection. (B) Expression of miR-301a during caerulein-induced chronic pancreatitis in WT mice (n = 5 per group). Pancreatic tissues were harvested at 6 weeks after the first caerulein or saline (control) injection. (C) Expression of miR-301a in caerulein accelerated PanIN formation in *Pdx1-Cre*;*Kras*^{G12D} mice (n = 5 per group). Pancreatic tissues were harvested at day 0 (d0), day 1 (d1), day 7 (d7), and day 21 (d21) after the last caerulein injection. (D) Expression of miR-301a in Kras-driven PanIN spontaneous progression in *Pdx1-Cre*;*Kras*^{G12D} mice at 9, 13, or 18 weeks of age. The pancreatic tissues (n = 6 per group) were harvested from *LSL-Kras*^{G12D} mice at 9 weeks of age and from *Pdx1-Cre*;*Kras*^{G12D} mice at 9, 13, or 18 weeks of age. Total RNA was extracted from pancreatic tissues, and miR-301a expression was quantified by real-time PCR. (E) Relative expression of miR-301a in serum from patients with chronic pancreatitis. (F) IL-6 concentration was measured by ELISA in serum from patients with chronic pancreatitis. (G) TGF-β concentration was measured by ELISA in serum from patients with chronic pancreatitis. Healthy controls, n = 49; pancreatitis patients, n = 75. (H) Spearman's rank correlation analysis was performed to compare the serum levels of IL-6 and miR-301a expression in patients with chronic pancreatitis (r = 0.3112, p = 0.0247). (I) Spearman's rank correlation analysis was performed to compare the serum levels of TGF-β and miR-301a expression in patients with chronic pancreatitis (r = 0.2594, p = 0.0439). Values are mean ± SD. **p < 0.01, *p < 0.05 indicate significant difference between the indicated groups.

with WT mice, as demonstrated by a decrease in the percentage of lesion area on H&E and Masson trichrome (blue) staining (Figure 2B). The expression level of α-smooth muscle actin (α-SMA), a marker of myofibroblasts, was markedly reduced in *miR-301a*$^{-/-}$ mice (Figure 2C). Given that activation of PSCs has been regarded as the core event in the development of pancreatic fibrosis, we quantified the number of desmin^{+} and GFAP^{+} cells (desmin and GFAP are specific markers of PSCs). Numbers of desmin^{+} and GFAP^{+} cells were significantly reduced in *miR-301a*$^{-/-}$ mice compared with their WT counterparts (Figures 2D and 2E). In agreement with their reduced pancreatic injury, *miR-301a*$^{-/-}$ mice also showed significantly reduced cytokeratin-19 (KRT19) positive areas compared with their WT littermates (Figure 2F). However, the expression levels of the cytokines IL-6, TGF-β, interferon-γ, IL-17A, and IL-8 were not significantly different between groups (Figure S2A). Notably, Stat3 activation was slightly increased in pancreatic tissue from *miR-301a*$^{-/-}$ mice treated with caerulein compared with that in WT mice, as assessed by increased phosphorylation of Stat3, which suggests that Stat3-driven inflammation is not an essential underlying cause of the phenotypic differences between *miR-301a*$^{-/-}$ mice and WT mice (Figure S2B). Taken together, these results suggest that

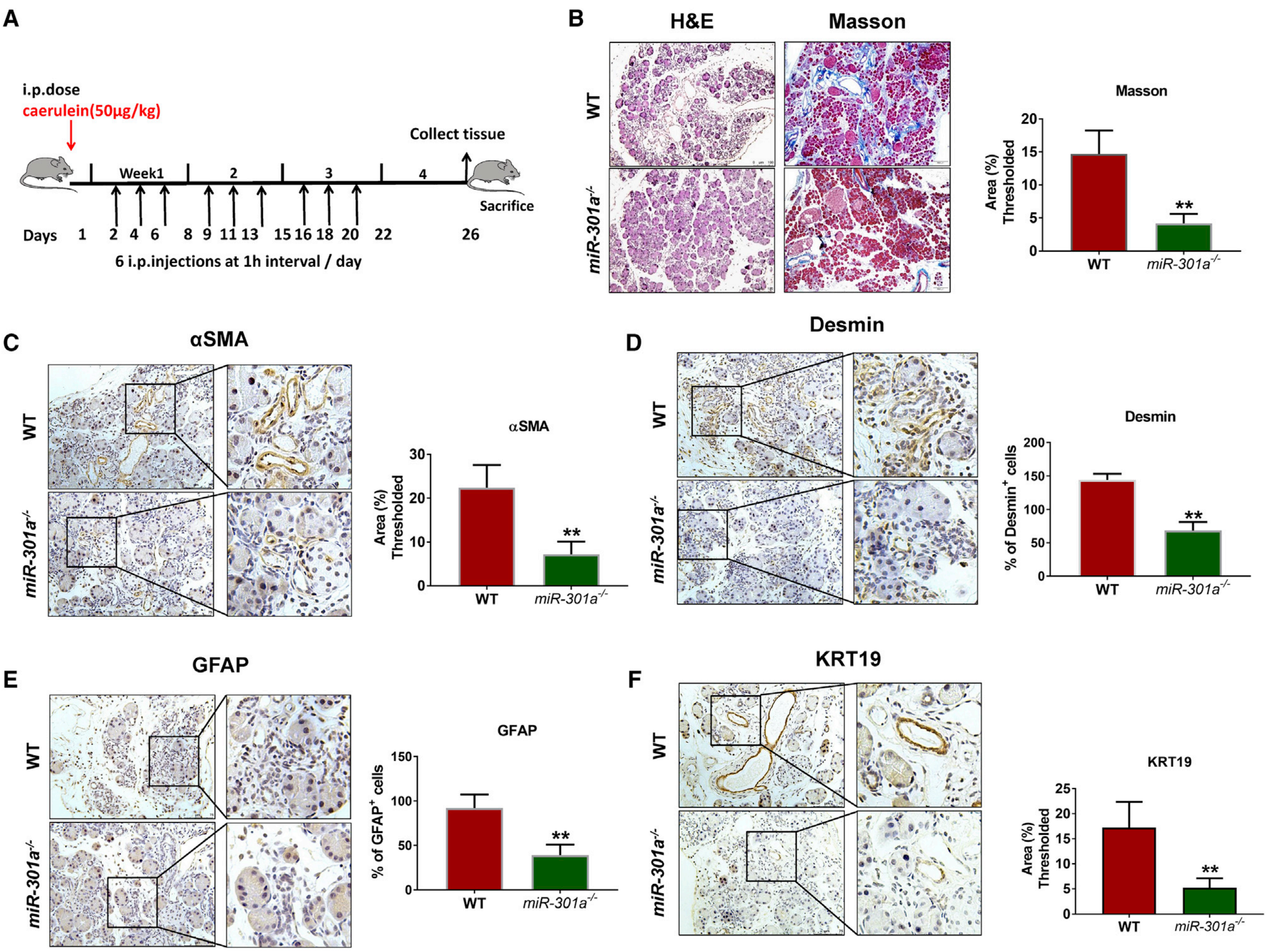

Figure 2. Response of WT and *miR-301a*$^{-/-}$ mice to caerulein-induced chronic pancreatitis

(A) Schematic protocol of the caerulein-induced chronic pancreatitis. (B) Left: representative histological sections of mouse pancreata stained with H&E from caerulein-treated WT (n = 8) and *miR-301a*$^{-/-}$ (n = 8) mice. Scale bars, 100 μm. Right: Masson staining showed a large increase in fibrosis in caerulein-treated WT and *miR-301a*$^{-/-}$ mice. Scale bars, 200 μm. (C–E) Immunohistochemical staining of α-SMA (C), desmin (D), and GFAP (E) showed activation of PSCs and increased fibrogenesis of pancreata from caerulein-treated WT (n = 8) and *miR-301a*$^{-/-}$ (n = 8) mice. Scale bars, 75 μm. (F) Immunohistochemical staining of KRT19 showed strong expression of intact pancreatic ducts over the time course of chronic pancreatitis in caerulein-treated WT (n = 8) and *miR-301a*$^{-/-}$ (n = 8) mice. Scale bars, 75 μm. Values are mean ± SD. **p < 0.01, *p < 0.05 indicate significant difference between caerulein-treated WT and *miR-301a*$^{-/-}$ groups.

miR-301a deficiency reduced the severity of pancreatic fibrosis and has a protective role in chronic pancreatitis versus acute pancreatitis in mice.

miR-301a deletion protects against spontaneous and pancreatitis-accelerated PanIN formation

Given the role of miR-301a in tissue fibrosis and inflammatory-driven tumorigenesis, we hypothesized that miR-301a deletion in mice would alleviate the PanIN lesion by reducing myofibroblast accumulation. To study the role of miR-301a in pancreatic ductal adenocarcinoma precursor formation, we crossed *miR-301a*$^{-/-}$ mice with *Pdx1-Cre*;*Kras*^{G12D} mice. Alcian blue staining revealed a marked decrease of intestinal mucin accumulation in *miR-301a*$^{-/-}$;*Pdx1-Cre*;*Kras*^{G12D} mice compared with *Pdx1-Cre*;*Kras*^{G12D} mice at the age of 9 weeks (Figures 3A and 3B). At 18 weeks old, the pancreata from *miR-301a*$^{-/-}$;*Pdx1-Cre*;*Kras*^{G12D} mice was significantly smaller and had less body weight than that from *Pdx1-Cre*;*Kras*^{G12D} mice (Figures 3C and 3D). Histological changes of the pancreas were assessed via H&E, Alcian blue, and Masson stainings. As anticipated, the extent of pancreatic tissue injury of *miR-301a*$^{-/-}$;*Pdx1-Cre*;*Kras*^{G12D} mice was significantly lower than that of *Pdx1-Cre*;*Kras*^{G12D} mice (Figures 3E–3G).

Next, we examined the effect of miR-301a deletion on pancreatitis-accelerated PanIN formation. Seven days after caerulein injection, the exocrine compartment of *Pdx1-Cre*;*Kras*^{G12D} mice had been replaced by fibrosis and ductal structures. In contrast, *miR-301a*$^{-/-}$;*Pdx1-Cre*;*Kras*^{G12D} mice exhibited dramatically less pancreatic injury and proliferation of pancreatic ductal epithelial cells (Figures 3H–3K).

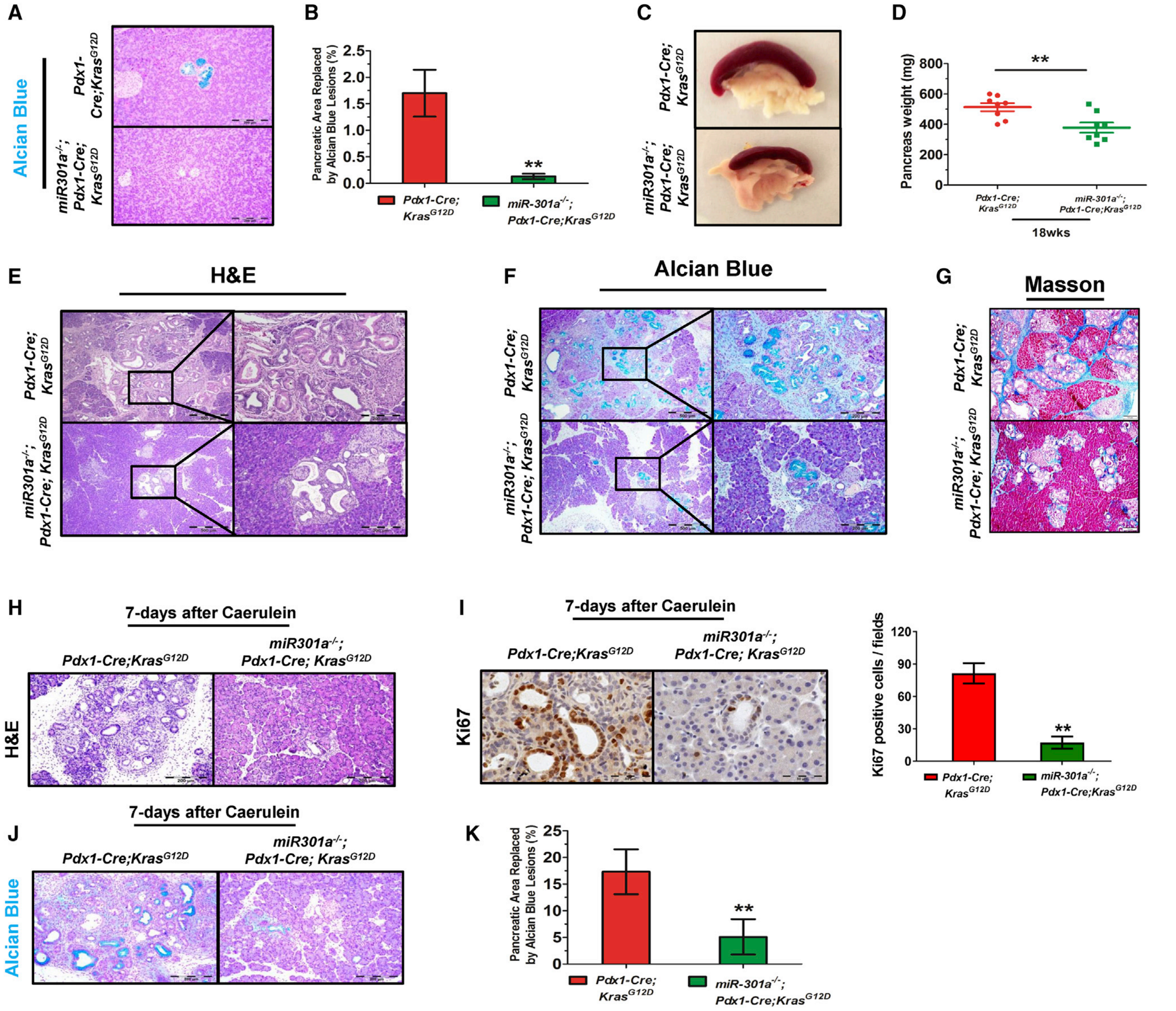

Figure 3. miR-301a deletion protects against spontaneous and pancreatitis-accelerated PanIN formation

(A) Alcian blue staining shows a spontaneous PanIN lesion in 9-week-old mice. Scale bars, 200 μm. (B) Alcian blue-positive PanIN lesion size compared with the whole pancreas. (C) and (D) Representative gross images (C) and weight pancreas (D) isolated from *Pdx1-Cre*;*Kras*^{G12D} (n = 8) and *miR-301a*$^{-/-}$;*Pdx1-Cre*;*Kras*^{G12D} (n = 8) littermate mice at 18 weeks of age. (E) H&E histological sections of pancreas isolated from *Pdx1-Cre*;*Kras*^{G12D} (n = 8) and *miR-301a*$^{-/-}$;*Pdx1-Cre*;*Kras*^{G12D} (n = 8) littermate mice at 18 weeks of age. (F) Alcian blue-positive PanIN lesion in total pancreas isolated from *Pdx1-Cre*;*Kras*^{G12D} (n = 8) and *miR-301a*$^{-/-}$;*Pdx1-Cre*;*Kras*^{G12D} (n = 8) littermate mice at 18 weeks of age. (G) Masson staining shows a large increase in fibrosis in PanIN lesion in total pancreas isolated from *Pdx1-Cre*;*Kras*^{G12D} (n = 8) and *miR-301a*$^{-/-}$;*Pdx1-Cre*;*Kras*^{G12D} (n = 8) littermate mice at 18 weeks of age. Scale bars, 200 μm. (H) H&E histological sections of pancreas from 9-week-old *Pdx1-Cre*;*Kras*^{G12D} and *miR-301a*$^{-/-}$;*Pdx1-Cre*;*Kras*^{G12D} mice 7 days after caerulein treatment. Scale bars, 200 μm. (I) Cell proliferation as determined by Ki67 staining. Scale bars, 50 μm. (J) Alcian blue staining shows PanIN lesions in 9-week-old mice 7 days after caerulein treatment. Scale bars, 200 μm. (K) Alcian blue-positive PanIN lesion size compared with the whole pancreas. **p ≤ 0.01 indicates significance of the differences between the indicated groups.

Furthermore, the expression of α-SMA, desmin, and GFAP was significantly downregulated in the pancreas from *miR-301a*$^{-/-}$;*Pdx1-Cre*; *Kras*^{G12D} mice compared with *Pdx1-Cre*; *Kras*^{G12D} mice (Figure S3A). The expression of IL-6, TNF-α, IL-17A, IL-1α, and IL-1β was significantly lower in pancreatic tissues from *miR-301a*$^{-/-}$;*Pdx1-Cre*; *Kras*^{G12D} mice compared with *Pdx1-Cre*;*Kras*^{G12D} mice (Figure S3B).

Transcriptional signature analysis of pancreatic tissues from PanIN mice

To investigate the molecular pathways underlying the effect of miR-301a deletion in PanIN formation, we performed RNA sequencing (RNA-seq) using total RNA isolated from pancreatic tissues of *Pdx1-Cre*;*Kras*^{G12D} and *miR-301a*$^{-/-}$;

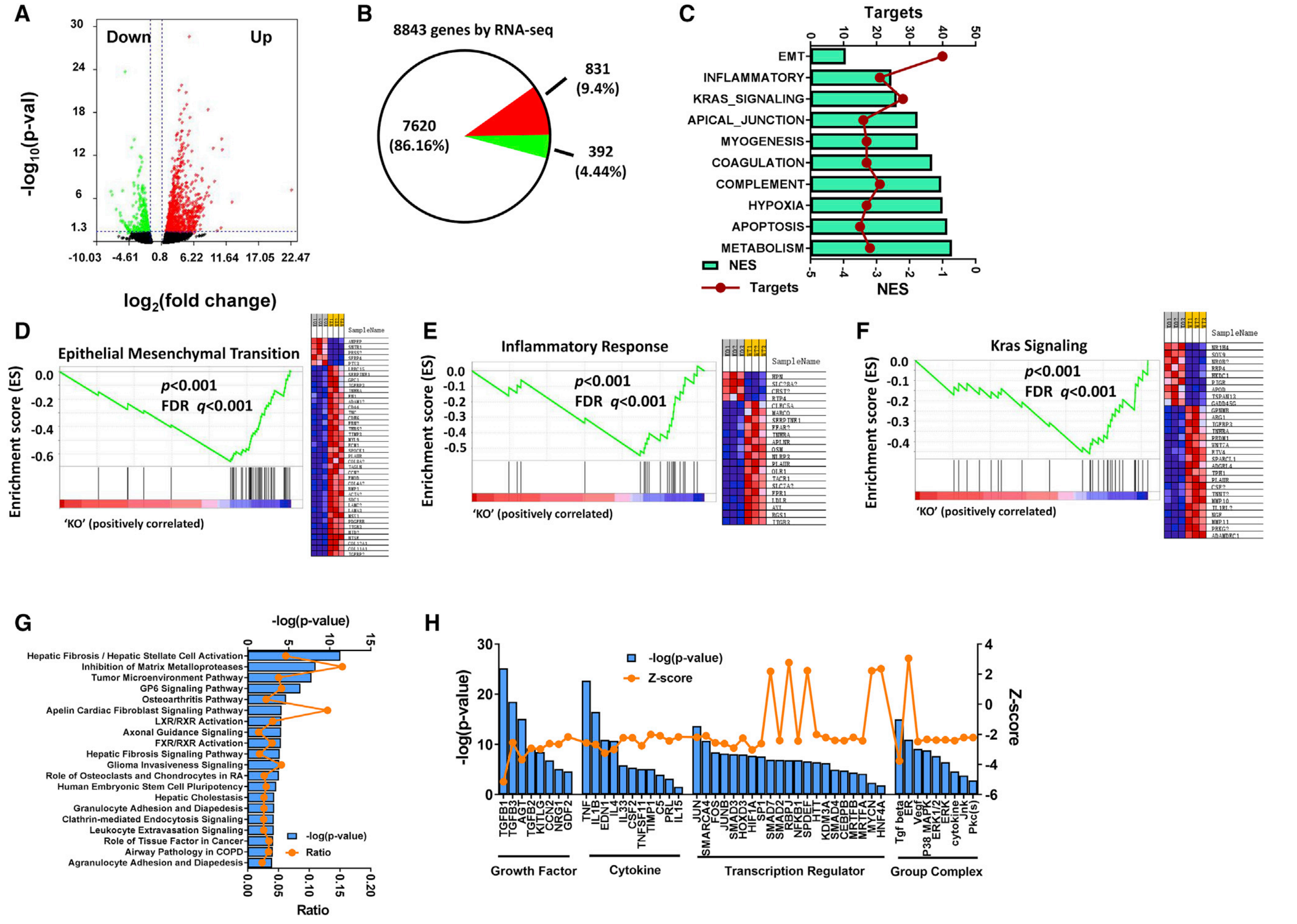

Figure 4. Molecular pathways perturbed by miR-301a deletion in PanIN formation

(A) Volcano plot shows differentially regulated gene expression from RNA-seq analysis in pancreas from 9-week-old *Pdx1-Cre*;*Kras*^{G12D} and *miR-301a*$^{-/-}$;*Pdx1-Cre*;*Kras*^{G12D} mice 7 days after caerulein treatment (n = 3 per group). (B) RNA-seq revealed a total of 8,843 genes, of which 831 genes were upregulated and 392 were downregulated. (C) GSEA analysis of the enrichment pathways with normalized enrichment score (NES) and targets. (D–F) Individual GSEA plots for the top three significant pathways including epithelial mesenchymal transition (D), inflammatory response (E), and Kras signaling (F) gene sets are shown. (G) IPA analysis of canonical signaling pathways from above gene sets. (H) IPA analysis of upstream factors from the above gene sets.

Pdx1-Cre;*Kras*^{G12D} mice 7 days after caerulein treatment. We identified a total of 1,223 differentially expressed genes (DEGs), including 831 upregulated DEGs and 392 downregulated DEGs, in pancreata from *Pdx1-Cre*;*Kras*^{G12D} and *miR-301a*$^{-/-}$;*Pdx1-Cre*;*Kras*^{G12D} mice (Figures 4A and 4B). Gene set enrichment analysis (GSEA) identified a significant decrease in three pathways upon miR-301a deletion—epithelial mesenchymal transition, inflammatory response, and Kras signaling—which suggest a critical role for miR-301a in tumor-stroma crosstalk (Figures 4C–4F).

To explore changes in molecular and upstream signaling upon miR-301a deletion, we used the Ingenuity Pathway Analysis (IPA) software in conjunction with the gene sets described above. The IPA results revealed that canonical pathways involved in stellate cell activation, inhibition of matrix metalloproteases, and tumor microenvironment pathway were enriched (Figure 4G). As stellate cells are activated by growth factors, cytokines, transcription regulators, and intracellular molecules, we set out to examine how these activators differed in pancreatic tumorigenesis in *miR-301a*$^{-/-}$;*Pdx1-Cre*; *Kras*^{G12D} mice versus *Pdx1-Cre*;*Kras*^{G12D} mice. As shown in Figure 4H, activation *Z* scores of all growth factors and cytokines were reduced in *miR-301a*$^{-/-}$;*Pdx1-Cre*;*Kras*^{G12D} mice compared with *Pdx1-Cre*;*Kras*^{G12D} mice, with TGF-β being the most significant change in both transcription regulator and group complex. Taken together, our data support a role of miR-301a anti-fibrotic response in pancreatic tumorigenesis via the TGF-β pathway.

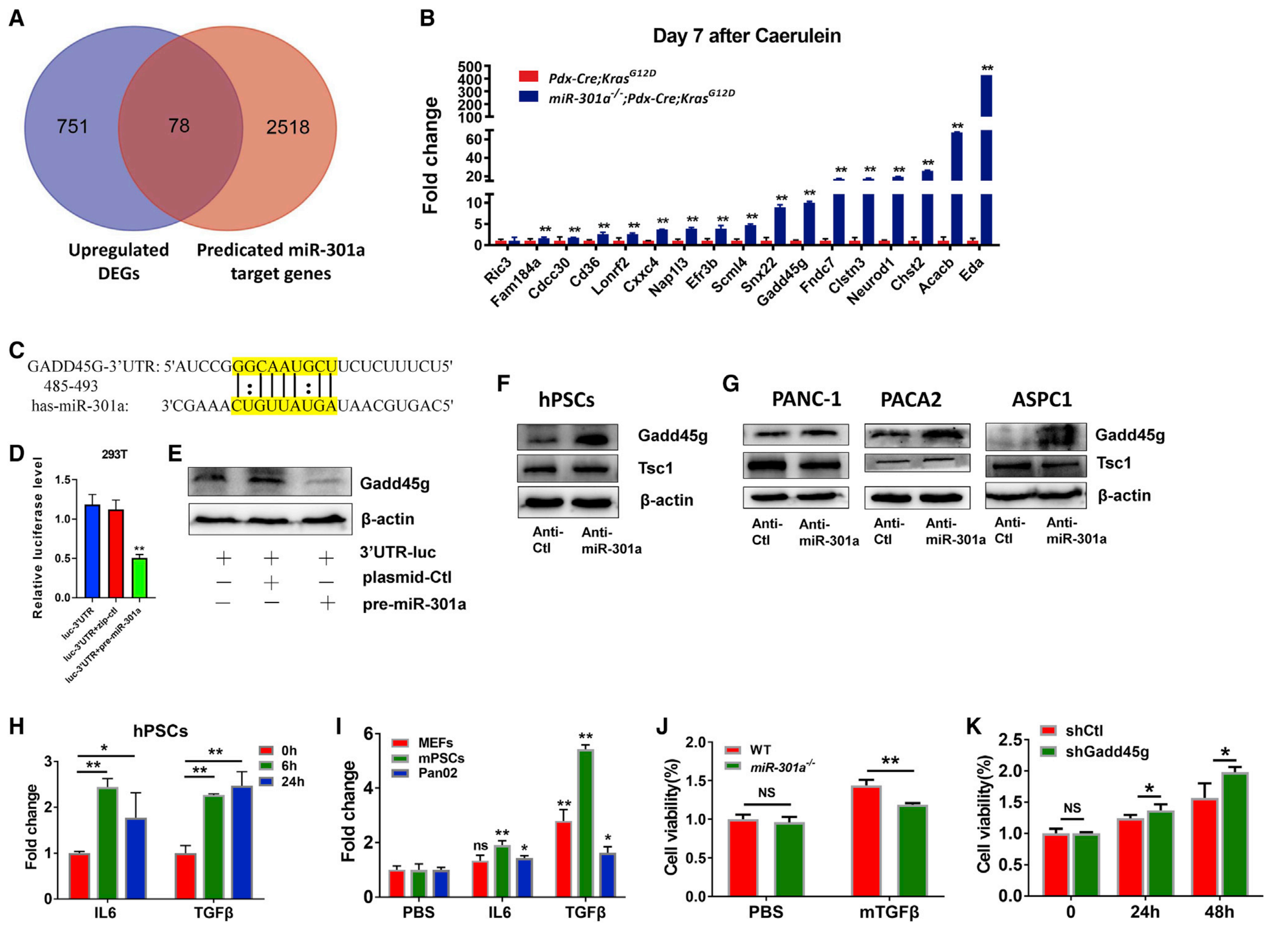

Figure 5. Inhibition of PanIN formation in *miR-301a*$^{-/-}$;*Pdx1-Cre*;*Kras*^{G12D} mice correlates with elevated Gadd45g expression and reduced PSCs proliferation

(A) Venn diagram of specific genes between predicted miR-301a targets from miRWalk database and DEGs identified by RNA-seq. (B) Real-time PCR validation of 17 significantly upregulated genes from (A). (C) Prediction of major interference sites between miR-301a and the Gadd45g mRNA 3′ UTR using TargetScan. (D) Luciferase activity in 293T cells transfected with the indicated luciferase reporter with either a control plasmid or a precursor miR-301a plasmid. (E) Western blot analysis of Gadd45g expression in 293T cells with the indicated luciferase reporter with either a control plasmid or a precursor miR-301a plasmid. (F) Western blot analysis of Gadd45g and Tsc1 expression in human PSCs transfected with anti-control (Anti-Ctl) or LNA-anti-miR-301a (Anti-miR-301a) oligonucleotide. (G) Western blot analysis of Gadd45g and Tsc1 expression in human PSCs transfected with anti-control (Anti-Ctl) or LNA-anti-miR-301a (Anti-miR-301a) oligonucleotide. (H) Expression of miR-301a in hPSCs treated with IL-6 or TGF-β. (I) Expression of miR-301a in mouse embryonic fibroblasts (MEFs), mouse PSCs (mPSCs), or Pan02 cells treated with IL-6 or TGF-β. (J) Cell viability of WT and *miR-301a*$^{-/-}$ PSCs treated with TGF-β. (K) Cell viability of *miR-301a*$^{-/-}$ PSCs transfected with shRNA-control (shCtl) or shRNA-Gadd45g (shGadd45g) and treated with TGF-β at the indicated time. Values are mean ± SD. $^{**}p < 0.01$, $^{*}p < 0.05$ indicate significant difference between the indicated groups. NS, not significant.

Inhibition of PanIN formation in *miR-301a*$^{-/-}$;*Pdx1-Cre*; *Kras*^{G12D} mice correlates with elevated Gadd45g and Tsc1 expression and inhibition of PSC proliferation

Our previous work demonstrated that Tsc1 was a direct target of miR-301a and was involved in pulmonary fibrosis.[12] Here, we measured Tsc1 expression in both chronic pancreatitis and PanIN mice models. In agreement with previous results, Tsc1 was significantly upregulated in *miR-301a*$^{-/-}$ mice and *miR-301a*$^{-/-}$;*Pdx1-Cre*;*Kras*^{G12D} mice treated with caerulein compared with their littermates (Figures S4A–S4C). Similarly, mTOR activation and the expression levels of two fibrosis markers, α-SMA and fibronectin, showed remarkably lower expression levels in fibrotic pancreatic tissues when miR-301a was deleted (Figures S4A and S4B). Furthermore, significantly lower levels of α-SMA and fibronectin were found in miR-301a deleted mouse PSCs treated with TGF-β (Figure S4D). These results further confirm a key role of the miR-301a/Tsc1/mTOR signaling pathway in pancreatitis and pancreatitis-driven tumorigenesis.

To probe the new targets of miR-301a in PanIN lesions, we compared the list of all upregulated DEGs identified from pancreatic tissues in *Pdx1-Cre*;*Kras*^{G12D} and *miR-301a*$^{-/-}$;*Pdx1-Cre*;*Kras*^{G12D} mice with the previously reported 2,596 miR-301a predicted target genes.[33] We found an overlap of 78 genes between upregulated DEGs and predicted miR-301a target genes (Figure 5A). Based on the reads per

kilobase of exon per million mapped reads (RPKM) value ($p < 0.05$) and $\log_2$(fold change) > 1, we choose 17 genes which were all significantly upregulated in pancreatic tissues from *miR-301a*$^{-/-}$;*Pdx1-Cre*;*Kras*^{G12D} mice and correlated with poor prognosis in patients with pancreatic cancer (Figure S5A). Upregulation of these 17 genes was further verified by quantitative real-time PCR (qPCR) (Figure 5B). We focused on Gadd45g, given its known function in cellular growth arrest and DNA damage, and investigated whether Gadd45g was a direct target gene of miR-301a. miRNA-target prediction analysis revealed a major binding site for miR-301a within the Gadd45g RNA 3′ UTR (Figure 5C). Results from the luciferase reporter assay showed that miR-301a was directly bound to Gadd45g mRNA and that Gadd45g expression was downregulated in 293T cells (Figures 5D and 5E). Next, we evaluated Gadd45g and Tsc1 expression in human PSCs and cancer cell lines (PANC-1, PACA2, and ASPC1). Gadd45g expression was significantly upregulated in stellate cells and cancer cells when miR-301a was inhibited, whereas Tsc1 expression was not altered (Figures 5F and 5G). In addition, a positive correlation between Tsc1 and Gadd45g expression was found in pancreatic cancer patients from The Cancer Genome Atlas (TCGA) dataset (Figure S6A).

Given the well-established role of PSCs in pancreatic fibrogenesis and tumorigenesis, we wondered whether Gadd45g upregulation in PSCs upon miR-301a inhibition led to cell growth arrest. First, we found miR-301a was induced in both human and mouse PSCs after TGF-β or IL-6 treatment as well as in mouse embryonic fibroblast and pancreatic cancer cells (Figures 5H and 5I). Second, we isolated PSCs from mice and showed that TGF-β induced significant cell growth in both WT and *miR-301a*$^{-/-}$ PSCs while it significantly reduced PSC proliferation in *miR-301a*$^{-/-}$ PSCs compared with WT PSCs (Figure 5J). As expected, concomitant knockdown of Gadd45g in *miR-301a*$^{-/-}$ PSCs reversed the reduced cellular proliferation mediated by miR-301a deletion in PSCs (Figure 5K).

Inhibition of Stat3 activation in PanIN and PSCs correlates with miR-301a and Gadd45g

To better understand how miR-301a deletion leads to inhibition of Stat3 activation in PanIN and PSCs, we first measured the levels of Stat3 activation. Consistent with previous reports, significantly less Stat3 activation was found in *miR-301a*$^{-/-}$;*Pdx1-Cre*;*Kras*^{G12D} pancreatic lesion tissues compared with *Pdx1-Cre*;*Kras*^{G12D} pancreatic tissues (Figure 6A), with such activation being markedly inhibited mainly in ductal and stroma cells (Figure 6B). A marked increase of Gadd45g expression in pancreatic stroma cells was observed in *miR-301a*$^{-/-}$;*Pdx1-Cre*;*Kras*^{G12D} mice (Figure 6C). Our previous study showed that miR-301a regulates Stat3 activation in different types of cells.[26] Therefore, we further examined Stat3 activation and Gadd45g expression in a human pancreatic stellate cell line (hPSCs) with miR-301a inhibition. As expected, Stat3 was constitutively activated in hPSCs treated with IL-6 or TGF-β; however, significantly less Stat3 activation was found in hPSCs in conditions of miR-301a inhibition. Accordingly, Gadd45g was highly expressed in miR-301a knockdown hPSCs (Figures 6D and 6E).

As Gadd45g-induced senescence in hepatocellular carcinoma cells depends on the activation of Stat3,[27] we sought to determine whether an increase of Gadd45g reduced cell proliferation by repressing Stat3 activity. Stat3 activation was significantly reduced when anti-miR-301a was introduced into hPSCs, whereas concomitant Gadd45g knockdown reversed the Stat3 activation (Figure 6F). It has been reported that the supernatant of PSCs can stimulate tumor cell proliferation. Therefore, we explored whether miR-301a deletion in PSCs can reduce Stat3 activation in pancreatic cancer cells. Pan02, a murine pancreatic cancer cell line, was treated with the supernatant of PSCs isolated from WT and *miR-301a*$^{-/-}$ mice. We found that Stat3 activation was significantly reduced in Pan02 cells treated with supernatant from *miR-301a*$^{-/-}$ PSCs compared with cells treated with supernatant from WT cells (Figure 6G). Similar results were observed in the human pancreatic cancer cell PANC-1 when treated with the supernatant from hPSCs transfected with anti-Ctl or anti-miR-301a (Figure 6H). Furthermore, based on TCGA data, a negative correlation between Gadd45g and Stat3 expression was found in pancreatic cancer patients ($r = -0.197$, $p = 0.00831$; Figure 6I). Although Gadd45g appears to be downregulated in many cancers, we found no significant differences in Gadd45g expression in pancreatic cancer tissues versus normal tissues (Figure 6J). However, significant correlations between Gadd45g expression and patient survival were from TCGA data analysis, further suggesting its key role in hPSCs or other stroma cells during pancreatic tumorigenesis (Figure 6K). Collectively, these data indicate that miR-301a/Gadd45g might have a general impact on PSC fate in pancreatic tumorigenesis, and provide compelling evidence that elevated Gadd45g expression contributes to attenuated tumorigenesis by modulating tumor-PSC crosstalk.

DISCUSSION

Pancreatitis is a high-risk factor for pancreatic cancer. A large cohort study reported that 14.8% patients with pancreatic cancer had a prior diagnoses of pancreatitis.[34] However, the relationship between pancreatitis and pancreatic cancer has not been completely elucidated. In the present study, we reveal a role for a pro-inflammatory miRNA, miR-301a, in pancreatitis and pancreatitis-associated PanIN, which are conditions known to accelerate the development of pancreatic ductal adenocarcinoma. miR-301a is also known to be activated by multiple inflammatory factors in both acute pancreatitis and chronic pancreatitis. The major findings of this study are that miR-301a regulates the activation of PSCs and that miR-301a deletion reduces pancreatic fibrosis induced by caerulein in mouse models. In mice with acute pancreatitis, deletion of miR-301a was not protective; however, in contrast, deletion of miR-301a significantly reduced fibrosis in chronic pancreatitis, suggesting a function of miR-301a in repetitive and persistent inflammation rather than in acute tissue inflammatory injury. Moreover, deletion of miR-301a reduced the characteristic features of fibrosis in spontaneous and pancreatitis-accelerated PanIN formation in *Pdx1-Cre*;*Kras*^{G12D} mice. We also demonstrate that the protective role of miR-301a deletion in chronic pancreatitis and PanIN is dependent on two downstream axes: Tsc1/mTOR and Gadd45g/Stat3. Of note, aberrant

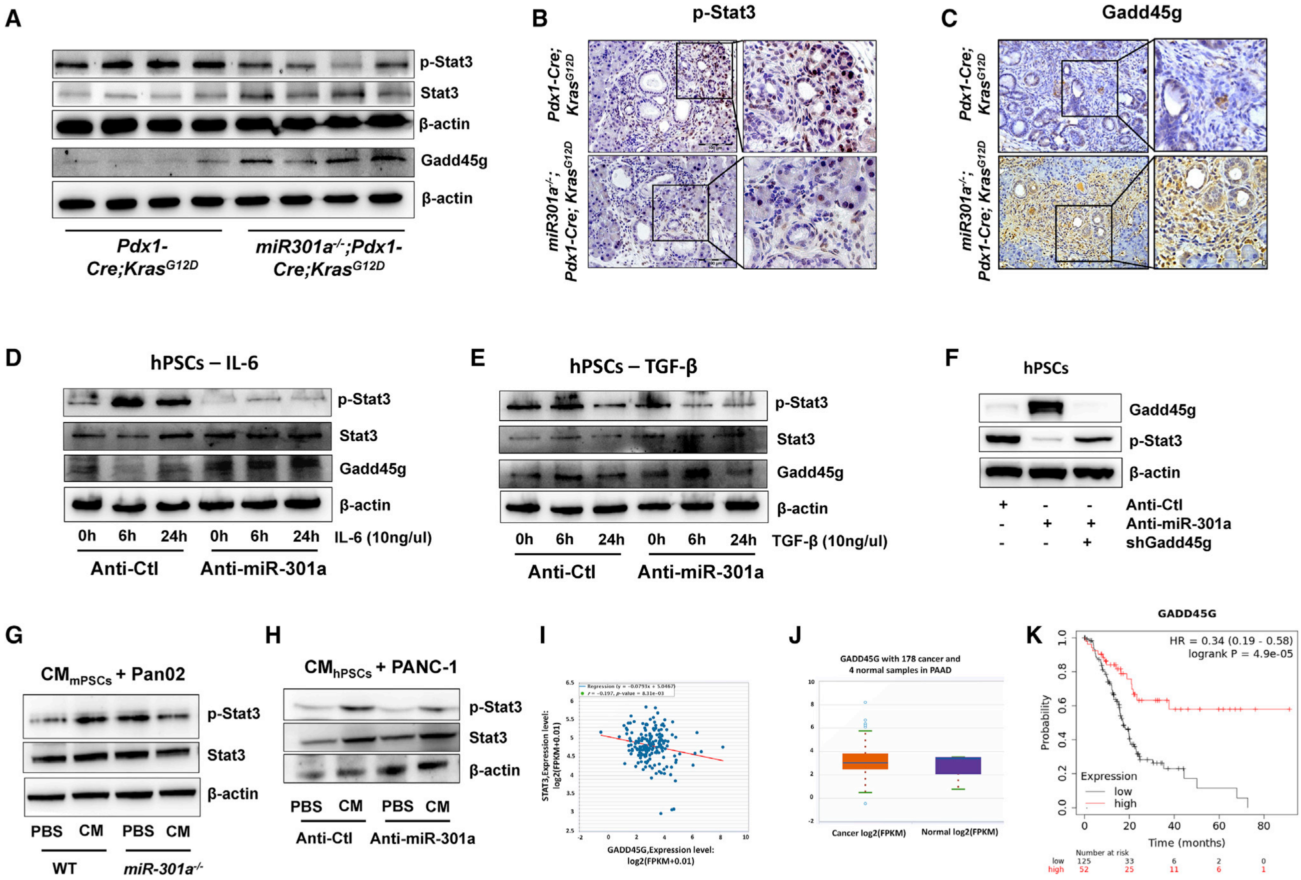

Figure 6. Inhibition of Stat3 activation in PanIN and PSCs correlates with miR-301a and Gadd45g

(A) Western blot shows the expression of p-Stat3, Stat3, and Gadd45g in pancreatic tissues from 9-week-old *Pdx1-Cre*;*Kras*^{G12D} (n = 4) and *miR-301a*$^{-/-}$;*Pdx1-Cre*;*Kras*^{G12D} (n = 4) mice 7 days after caerulein treatment. (B and C) Immunohistochemical staining of phosphor Stat3 (B) and Gadd45g (C) in pancreatic tissues from *Pdx1-Cre*;*Kras*^{G12D} (n = 8) and *miR-301a*$^{-/-}$;*Pdx1-Cre*;*Kras*^{G12D} (n = 8) littermate mice 7 days after caerulein treatment. Scale bars, 100 μm. (D and E) Human PSCs were transfected with anti-Ctl and anti-miR-301a and then were treated with IL-6 (D) or TGF-β (E). The expression of p-Stat3, total Stat3, and Gadd45g were measured by western blot. (F) Human PSCs were transfected with the indicated shRNA, including anti-Ctl or anti-miR-301a with or without shRNA-Gadd45g. The expression of p-Stat3 and Gadd45g were measured by western blot. (G) Pan02 cells were treated with conditional medium collected from WT and *miR-301a*$^{-/-}$ PSCs. The activation of Stat3 was evaluated by western blot. (H) PANC-1 cells were treated with conditional medium collected from human PSCs transfected with anti-Ctl or anti-miR-301a oligonucleotide. The activation of Stat3 was evaluated by western blot. (I) Correlation analyses between Gadd45g and Stat3 in pancreatic cancer patients in TCGA database. (J) Gadd45g expression in human pancreatic cancer samples compared with normal samples in TCGA database. (K) Kaplan-Meier analysis (log-rank test) of overall survival of human pancreatic cancer samples from Kaplan-Meier plotter database based on the expression of Gadd45g. **p < 0.01, *p < 0.05 indicate significant difference between normal tissue and tumor tissue.

regulation of Gadd45g/Stat3 was only found to accelerate PanIN formation but not chronic pancreatitis in mice ablated of miR-301a. These studies infer the complexity of miR-301a regulation in pancreatic stromal cells in PanIN in conditions where oncogenic Kras alleles are expressed in pancreatic epithelial cells, and suggest that miR-301a could be a therapeutic target for preventing pathological tissue fibrosis.

miR-301a is highly upregulated in fibrotic lung and liver,[35–37] which highlights the potential diagnostic and therapeutic implications of this miRNA in regulating fibrogenic mediators such as TGF-β and IL-6. Previously, we reported that miR-301a was upregulated in fibrotic lung tissues from mice and idiopathic pulmonary fibrosis.[12] Expression of miR-130a/301a in serum has been proposed as a distinct biomarker in chronic pancreatitis patients.[38] In our study, we found that miR-301a was significantly upregulated in pancreatic lesions during conditions of short and persistent inflammation in mice. In addition, we found a significant positive correlation between miR-301a expression and the expression of TGF-β and IL-6 in serum from patients with chronic pancreatitis. Specifically, miR-301a was induced in PanIN progression and in early PDAC mice and patients, and marked fibrosis and myofibroblasts were found to be accumulated in stromal focus lesions. Overall, these data suggest that serum miR-301a, in combination with examination of TGF-β and IL-6, might be a fibrosis marker for fibrosis in patients with chronic pancreatitis and early pancreatic cancer.

Next, we demonstrated the potential therapeutic role of miR-301a in pancreatic fibrosis and Kras-driven intra-neoplasia fibrosis. Given the robust effect of miR-301a in activating NF-κB and Stat3 in the tumor microenvironment, we expected that deletion of miR-301a would lead to a reduction of expression of inflammatory cytokines in caerulein-induced chronic pancreatitis in mice. However, Stat3 activation was more pronounced in chronic pancreatic tissues when miR-301a was deleted, and alteration of expression levels of inflammatory cytokines was not observed. These data suggest that miR-301a/Stat3 axis might be necessary for progression of chronic pancreatitis and might hold potential in the treatment of pancreatic cancer. Our previous study reported that deletion of miR-301a protected mice from bleomycin-induced lung fibrosis by reducing mTOR activation in a Tsc1-dependent manner. We found a similar effect for the miR-301a/Tsc1/mTOR axis in caerulein-induced pancreatic fibrosis, further confirming the key role of this axis signaling in pancreatic tissue fibrosis. Although Tsc1 expression was upregulated upon miR-301a inhibition in fibroblasts, there was no significant difference in Tsc1 expression either in PSCs or in pancreatic cancer cell lines when miR-301a was inhibited. Thus, we speculate that other miR-301a targets are involved in the regulation of PSCs and initiation of pancreatic cancer.

To dissect the mechanism of miR-301a action, we reasoned that the reduced fibrosis in chronic pancreatitis and PanIN in miR-301a-deficient mice is a result of impaired activation of PSCs. Our data show that miR-301a affects the proliferation of PSCs treated with TGF-β and that the number of PSCs in pancreatic tissue was significantly reduced in miR-301a-deficient mice. Next, we identified Gadd45g as a novel target gene of miR-301a in both PSCs and pancreatic cancer cell lines. Our results show a significant upregulation of Gadd45g in miR-301a-knockdown PSCs and miR-301a-deficient PanIN tissues. Moreover, increased Gadd45g expression led to inhibition of Stat3 activation in PSCs. It will therefore be interesting to examine whether Gadd45g is essential for PanIN initiation and pancreatic cancer formation. Notably, Stat3 activation was markedly downregulated in pancreatic cancer cells treated with the supernatant from PSCs in conditions of miR-301a inhibition. The role of stroma fibroblasts in pancreatic cancer is controversial, as these cells have been shown to either inhibit tumor progression or increase undifferentiation of pancreatic tumors. Blocking the communication between PSCs and pancreatic cancer cells by miR-301a depletion might lead to more effective therapies targeting the stroma.

In summary, the present findings further confirm the role of miR-301a/Tsc1 in tissue fibrosis. Our results describe key molecular signaling pathways, miR-301a/Gadd45g, which facilitate the direct interplay between PSCs and tumor cells. These findings might lead to the development of feasible therapeutic strategies in pancreatic cancer.

MATERIALS AND METHODS

Mice, cell lines, and patients

The generation of *miR-301a*$^{-/-}$ mice in the C57BL/6 × 129S hybrid background has been described previously.[26] *Pdx1-Cre*;*Kras*^{G12D} mice were purchased from the Jackson Laboratory and bred with *miR-301a*$^{-/-}$ mice to obtain *miR-301a*$^{-/-}$; *Pdx1-Cre*;*Kras*^{G12D} mice. For acute pancreatitis, mice received an 8-hourly intraperitoneal (i.p.) injection of caerulein (50 μg/kg, Sigma) for 1 day. To induce chronic pancreatitis, mice were administered 6-hourly i.p. injections per day, 3 days per week, for a total of 3 weeks. All mice were kept under pathogen-free conditions, and all experiments were performed under protocols approved by the Institutional Animal Care and Use Committee of Zhongshan City People's Hospital and The South China Normal University, China. Tissue samples included in this study were obtained from patients from the Cancer Research Institute of Zhongshan City, Zhongshan City People's Hospital, China. The study protocol was approved by the Clinical Research Ethics Committee of the Zhongshan City People's Hospital, China. Written informed consent was obtained from all participants before the start of the study. hPSCs were purchased from Sciencell, and pancreatic cancer cell lines were purchased from Shanghai Cell Bank of the Chinese Academy of Sciences. Mouse PSCs were isolated from pancreas of WT and *miR-301a*$^{-/-}$ mice as previously described.[39] All cells were cultured in Dulbecco's modified Eagle's medium supplemented with 10% fetal bovine serum (Gibco) and 1% penicillin/streptomycin.

Cell transfection and proliferation assay

Cells were transfected with LNA-anti-Control or LNA-anti-miR-301a by using Lipofectamine RNAiMAX reagent (Thermo Fisher Scientific). For knockdown of Gadd45g in cells, short hairpin RNA (shRNA)-Gadd45g (GAA AGC GCT GCA TGA GTT GCT) and nonsense sequence (shRNA-control, GGT GTG CAG TTG GAA TGT A) were synthesized and ligated into miRZip Lentivector-based anti-miRNA vector (System Biosciences). HEK-293T cells were seeded in a 6-well plate and transfected with packing plasmids and the shRNA constructs. Cells were cultured at 37°C for 48 h before harvesting. Target cells were infected with the lentivirus in the presence of 8 μg/mL of polybrene; medium was changed 6 h post infection. For the proliferation assay, cells were seeded in 96-well plates and treated with TGF-β at the indicated times. Cell proliferation was determined using the Cell Counting Kit-8 (Dojindo Molecular Technologies) following the manufacturer's protocol.

RNA sequencing and data processing

Total RNA was harvested using TRIzol reagent. Poly(A) RNAs were purified by using the Dynabeads mRNA purification kit, and poly(A) RNAs were fragmented at 94°C for 5 min before reverse transcription to cDNA with random primers. cDNA fragments were blunted and ligated to sequencing adapters. mRNA profiles were generated by deep sequencing using the Novaseq 6000 sequencing platform by Novogene China. Reads were mapped to the human reference genome (GRCh38) using STAR software (version 2.5.1), and the annotation from GENCODE version vM21 was employed. Differential gene expression was performed using DESeq2.[40]

DEG identification and bioinformatic analysis

The relative transcript abundance was measured in RPKM. DEGs were identified using an unpaired Student's t test with p value cutoff of 0.05 and fold change more than 2.0 (upregulation) or less than 0.5

(downregulation). GSEA was conducted using the software GSEA version 4.1.0. IPA software was used to identify enriched molecular pathways and upstream regulators. We considered both direct and indirect relationships that were experimentally observed and predicted in mouse species and used the "stringent" setting to filter molecules and relationships in tissues and cell lines. For canonical pathways, the −log(p value) was derived from the right-tailed Fisher's exact test. Transcription factors and cytokines were extracted from upstream regulator analysis. This analysis identifies causal molecules associated with differential expression using both the significance and direction of differential expression to specify causal predictions. The *Z* scores and p values based on overlap between predicted and observed regulator-regulated genes (Fisher's exact test) were calculated.[41]

miR-301a predicted target analysis

We used a comprehensive microRNA-target predicting database, miRWalk, which integrates six bioinformatics tools (Targetscan6.2, miRWalk 2.0, miRDB4.0, miRanda-rel2010, RNA22v2, and PITA) to predict the potential targets of miR-301a. The TargetScan online database was used to predict the major interference sites between miR-301a and the Gadd45g mRNA 3′ UTR.

Quantitative real-time PCR

To validate gene expression findings, we dissected pancreas tissues from mice, extracted total RNA using TRIzol (Invitrogen), and carried out reverse transcription using the iScript Reverse Transcription Supermix kit (Bio-Rad). The qPCR reaction contained 15 μL of SYBR Green mixture, 0.5 μL of forward primer, 0.5 μL of reverse primer, 5 μL of cDNA template, and 9 μL of H_2O. The SYBR Green mixture was optimized according to the amount of DNA polymerase, dNTP, reaction buffer, and dyes. The two-step method (95°C and 60°C) was used for thermocycling. qPCR was performed using the CFX96 real-time PCR detection system (Bio-Rad). qPCR primers are shown in Table S1. Each sample was analyzed in duplicate using β-actin as reference. qPCR for miR-301a detection was performed using the TaqMan assay (Thermo Fisher Scientific).

Luciferase reporter assay

The 3′ UTR sequence of Gadd45g was amplified and subcloned into the psiCHECK-2 dual luciferase reporter vector. HEK293T cells were then transfected with the psiCHEKC-2 plasmid containing pre-miR-301a or miR-control. After 48 h in culture, cells were lysed and the activities of firefly and Renilla luciferase were determined using a dual luciferase reporter assay system (GeneCopoeia).

Western blotting analysis

For western blotting analyses, pancreatic tissues or cell lines were lysed with RIPA buffer (Cell Signaling Technology) supplemented with protease inhibitor (Thermo Fisher Scientific). Proteins were then subjected to SDS-PAGE, transferred to polyvinylidene fluoride membranes, blocked with 5% nonfat dry milk for 1 h, and incubated with the primary antibodies at 4°C overnight. The primary antibodies used were Tsc1 (BA2879, BOSTER), phospho-mTOR-S2448 (381557, ZENBIO), Fibronectin (ab2413, Abcam), α-SMA (ab5694, Abcam), Gadd45g (A10286, Abclonal), Stat3 (9139, CST), p-Stat3-Tyr705 (9145, CST), β-actin (AC026, Abclonal). Following washes with TBST buffer, membranes were incubated with a horseradish peroxidase-conjugated anti-rabbit or anti-mouse secondary antibody for 1 h at room temperature. Protein immunoreactivity was visualized using the SuperSignal West Pico Chemiluminescent kit (Thermo Fisher Scientific). Pictures were scanned by automatic chemiluminescence imaging using the analysis system Tanon5200, and were acquired using PVCAM software (Tanon, China). All experiments were performed in triplicate.

Immunohistochemistry

Pancreatic tissues were fixed in 10% formalin solution, embedded in paraffin, and sectioned in 3-μm slices. Sections were stained with H&E and Masson's trichrome to detect the interstitial volume expansion. Antigen retrieval was performed in sodium citrate buffer at 96°C. Sections were washed with PBS three times, and endogenous peroxidase activity was blocked by incubating sections in a solution of 3% H_2O_2 for 5 min. Sections were then blocked with 10% normal goat serum. The primary antibodies used were α-SMA (ab5694, Abcam), Desmin (A3736, Abclonal), GFAP (A19058, Abclonal), KRT19 (A19040, Abclonal), Gadd45g (A10286, Abclonal), and Tsc1 (BA2879, BOSTER). Incubation with the primary antibody was performed at 4°C overnight. Tissue sections were developed using the Ultra Vision Detection System (Thermo Fisher Scientific).

ELISA

For determination of IL-6 and TGF-β secretion *ex vivo*, serum from clinical patients was collected. IL-6 and TGF-β concentrations were measured using ELISA kits (eBioscience) according to the manufacturer's protocol.

Data availability and correlation analysis

TCGA RNA-seq data for pancreatic cancer patients were downloaded and analyzed on starbase.sysu.edu.cn.[42] Survival plots of human pancreatic patients for 17 selected genes were evaluated on kmplot.com.[43]

Statistical analysis

Data are expressed as means ± SD. At least three independent experiments were performed. Results were subjected to statistical analysis using Student's t test for two-group comparisons or one-way ANOVA for multiple-group comparisons. Statistical significance was set at $p < 0.05$ or $p < 0.01$.

AVAILABILITY OF DATA AND MATERIALS

The data that support the findings of this study were submitted to the Gene Expression Omnibus database (accession number GEO: GSE176571, available from https://www.ncbi.nlm.nih.gov/geo/query/acc.cgi?acc=GSE176571).

SUPPLEMENTAL INFORMATION

Supplemental information can be found online at https://doi.org/10.1016/j.omtn.2022.01.011.

ACKNOWLEDGMENTS

The authors would like to thank all the reviewers who participated in the review and MJEditor (www.mjeditor.com) for its linguistic assistance during the preparation of the manuscript. This study was supported by grants from the Public Welfare Scientific Research Project of Zhongshan City (no. 2019B1015), the National Natural Science Foundation of China (nos. 82072697, 81773249, and 81772533), Key Area Research and Development Program of Guangdong Province (2019B030335001), and Guangzhou Science and Technology Plan Project (no. 202102010251).

AUTHOR CONTRIBUTIONS

F.L., M.W., and X.L. performed the experiments. Y.L. and K.C. participated in data analysis and animal experiments. X.W., M.Z., W.C., and X.T. assisted in immunostaining, western blot analysis, and data interpretation. P.W., M.J., and X.M. designed and wrote the manuscript. All authors reviewed the manuscript and approved the submission.

DECLARATION OF INTERESTS

The authors declare no competing interests.

REFERENCES

1. Omary, M.B., Lugea, A., Lowe, A.W., and Pandol, S.J. (2007). The pancreatic stellate cell: a star on the rise in pancreatic diseases. J. Clin. Invest. *117*, 50–59.

2. Apte, M.V., Haber, P.S., Applegate, T.L., Norton, I.D., McCaughan, G.W., Korsten, M.A., Pirola, R.C., and Wilson, J.S. (1998). Periacinar stellate shaped cells in rat pancreas: identification, isolation, and culture. Gut *43*, 128–133.

3. Bachem, M.G., Schneider, E., Gross, H., Weidenbach, H., Schmid, R.M., Menke, A., Siech, M., Beger, H., Grunert, A., and Adler, G. (1998). Identification, culture, and characterization of pancreatic stellate cells in rats and humans. Gastroenterology *115*, 421–432.

4. Ding, Z., Maubach, G., Masamune, A., and Zhuo, L. (2009). Glial fibrillary acidic protein promoter targets pancreatic stellate cells. Dig. Liver Dis. *41*, 229–236.

5. Masamune, A., Kikuta, K., Watanabe, T., Satoh, K., Hirota, M., Hamada, S., and Shimosegawa, T. (2009). Fibrinogen induces cytokine and collagen production in pancreatic stellate cells. Gut *58*, 550–559.

6. Apte, M., Pirola, R., and Wilson, J. (2011). The fibrosis of chronic pancreatitis: new insights into the role of pancreatic stellate cells. Antioxid. Redox Signal. *15*, 2711–2722.

7. Apte, M., Pirola, R., and Wilson, J. (2009). New insights into alcoholic pancreatitis and pancreatic cancer. J. Gastroenterol. Hepatol. *24*, S51–S56.

8. Xue, R., Jia, K., Wang, J., Yang, L., Wang, Y., Gao, L., and Hao, J. (2018). A rising star in pancreatic diseases: pancreatic stellate cells. Front. Physiol. *9*, 754.

9. Pang, T.C.Y., Wilson, J.S., and Apte, M.V. (2017). Pancreatic stellate cells: what's new? Curr. Opin. Gastroenterol. *33*, 366–373.

10. Gao, Z., Wang, X., Wu, K., Zhao, Y., and Hu, G. (2010). Pancreatic stellate cells increase the invasion of human pancreatic cancer cells through the stromal cell-derived factor-1/CXCR4 axis. Pancreatology *10*, 186–193.

11. Orozco, C.A., Martinez-Bosch, N., Guerrero, P.E., Vinaixa, J., Dalotto-Moreno, T., Iglesias, M., Moreno, M., Djurec, M., Poirier, F., Gabius, H.J., et al. (2018). Targeting galectin-1 inhibits pancreatic cancer progression by modulating tumor-stroma crosstalk. Proc. Natl. Acad. Sci. U S A *115*, E3769–E3778.

12. Wang, J., Li, X., Zhong, M., Wang, Y., Zou, L., Wang, M., Gong, X., Wang, X., Zhou, C., Ma, X., et al. (2020). miR-301a suppression within fibroblasts limits the progression of fibrosis through the TSC1/mTOR pathway. Mol. Ther. Nucleic Acids *21*, 217–228.

13. Jin, G., Hong, W., Guo, Y., Bai, Y., and Chen, B. (2020). Molecular mechanism of pancreatic stellate cells activation in chronic pancreatitis and pancreatic cancer. J. Cancer *11*, 1505–1515.

14. Ramakrishnan, P., Loh, W.M., Gopinath, S.C.B., Bonam, S.R., Fareez, I.M., Mac Guad, R., Sim, M.S., and Wu, Y.S. (2020). Selective phytochemicals targeting pancreatic stellate cells as new anti-fibrotic agents for chronic pancreatitis and pancreatic cancer. Acta Pharm. Sin. B *10*, 399–413.

15. Vogelmann, R., Ruf, D., Wagner, M., Adler, G., and Menke, A. (2001). Effects of fibrogenic mediators on the development of pancreatic fibrosis in a TGF-beta1 transgenic mouse model. Am. J. Physiol. Gastrointest. Liver Physiol. *280*, G164–G172.

16. Mace, T.A., Ameen, Z., Collins, A., Wojcik, S., Mair, M., Young, G.S., Fuchs, J.R., Eubank, T.D., Frankel, W.L., Bekaii-Saab, T., et al. (2013). Pancreatic cancer-associated stellate cells promote differentiation of myeloid-derived suppressor cells in a STAT3-dependent manner. Cancer Res. *73*, 3007–3018.

17. Lesina, M., Kurkowski, M.U., Ludes, K., Rose-John, S., Treiber, M., Kloppel, G., Yoshimura, A., Reindl, W., Sipos, B., Akira, S., et al. (2011). Stat3/Socs3 activation by IL-6 transsignaling promotes progression of pancreatic intraepithelial neoplasia and development of pancreatic cancer. Cancer Cell *19*, 456–469.

18. Fukuda, A., Wang, S.C., Morris, J.P., IV, Folias, A.E., Liou, A., Kim, G.E., Akira, S., Boucher, K.M., Firpo, M.A., Mulvihill, S.J., et al. (2011). Stat3 and MMP7 contribute to pancreatic ductal adenocarcinoma initiation and progression. Cancer Cell *19*, 441–455.

19. Shi, Y., Gao, W., Lytle, N.K., Huang, P., Yuan, X., Dann, A.M., Ridinger-Saison, M., DelGiorno, K.E., Antal, C.E., Liang, G., et al. (2019). Targeting LIF-mediated paracrine interaction for pancreatic cancer therapy and monitoring. Nature *569*, 131–135.

20. Miranda, K.C., Huynh, T., Tay, Y., Ang, Y.S., Tam, W.L., Thomson, A.M., Lim, B., and Rigoutsos, I. (2006). A pattern-based method for the identification of microRNA binding sites and their corresponding heteroduplexes. Cell *126*, 1203–1217.

21. Zhang, Y., Huang, B., Wang, H.Y., Chang, A., and Zheng, X.F.S. (2017). Emerging role of microRNAs in mTOR signaling. Cell Mol. Life Sci. *74*, 2613–2625.

22. Wang, Y., Liu, J., Liu, C., Naji, A., and Stoffers, D.A. (2013). MicroRNA-7 regulates the mTOR pathway and proliferation in adult pancreatic beta-cells. Diabetes *62*, 887–895.

23. Xu, K., Bian, D., Hao, L., Huang, F., Xu, M., Qin, J., and Liu, Y. (2017). microRNA-503 contribute to pancreatic beta cell dysfunction by targeting the mTOR pathway in gestational diabetes mellitus. EXCLI J. *16*, 1177–1187.

24. Jin, Y., Tymen, S.D., Chen, D., Fang, Z.J., Zhao, Y., Dragas, D., Dai, Y., Marucha, P.T., and Zhou, X. (2013). MicroRNA-99 family targets AKT/mTOR signaling pathway in dermal wound healing. PLoS One *8*, e64434.

25. Gismondi, A., Nanni, V., Monteleone, V., Colao, C., Di Marco, G., and Canini, A. (2021). Plant miR171 modulates mTOR pathway in HEK293 cells by targeting GNA12. Mol. Biol. Rep. *48*, 435–449.

26. Ma, X., Yan, F., Deng, Q., Li, F., Lu, Z., Liu, M., Wang, L., Conklin, D.J., McCracken, J., Srivastava, S., et al. (2015). Modulation of tumorigenesis by the pro-inflammatory microRNA miR-301a in mouse models of lung cancer and colorectal cancer. Cell Discov. *1*, 15005.

27. Zhang, L., Yang, Z., Ma, A., Qu, Y., Xia, S., Xu, D., Ge, C., Qiu, B., Xia, Q., Li, J., et al. (2014). Growth arrest and DNA damage 45G down-regulation contributes to Janus kinase/signal transducer and activator of transcription 3 activation and cellular senescence evasion in hepatocellular carcinoma. Hepatology *59*, 178–189.

28. Guo, D., Zhao, Y., Wang, N., You, N., Zhu, W., Zhang, P., Ren, Q., Yin, J., Cheng, T., and Ma, X. (2021). GADD45g acts as a novel tumor suppressor, and its activation suggests new combination regimens for the treatment of AML. Blood *138*, 464–479.

29. Thalheimer, F.B., Wingert, S., De Giacomo, P., Haetscher, N., Rehage, M., Brill, B., Theis, F.J., Hennighausen, L., Schroeder, T., and Rieger, M.A. (2014). Cytokine-regulated GADD45G induces differentiation and lineage selection in hematopoietic stem cells. Stem Cell Rep. *3*, 34–43.

30. Chi, H., Lu, B., Takekawa, M., Davis, R.J., and Flavell, R.A. (2004). GADD45beta/GADD45gamma and MEKK4 comprise a genetic pathway mediating STAT4-independent IFNgamma production in T cells. EMBO J. *23*, 1576–1586.

31. Ju, S., Zhu, Y., Liu, L., Dai, S., Li, C., Chen, E., He, Y., Zhang, X., and Lu, B. (2009). Gadd45b and Gadd45g are important for anti-tumor immune responses. Eur. J. Immunol. *39*, 3010–3018.

32. Scuto, A., Kirschbaum, M., Buettner, R., Kujawski, M., Cermak, J.M., Atadja, P., and Jove, R. (2013). SIRT1 activation enhances HDAC inhibition-mediated upregulation of GADD45G by repressing the binding of NF-kappaB/STAT3 complex to its promoter in malignant lymphoid cells. Cell Death Dis. *4*, e635.

33. Li, X., Zhong, M., Wang, J., Wang, L., Lin, Z., Cao, Z., Huang, Z., Zhang, F., Li, Y., Liu, M., et al. (2019). miR-301a promotes lung tumorigenesis by suppressing Runx3. Mol. Cancer *18*, 99.

34. Syed, A., Babich, O., Thakkar, P., Patel, A., Abdul-Baki, H., Farah, K., Morrissey, S., Mitre, M., Dhawan, M., Kochhar, G., et al. (2019). Defining pancreatitis as a risk factor for pancreatic cancer: the role, Incidence, and timeline of development. Pancreas *48*, 1098–1101.

35. Lu, L., Wang, J., Lu, H., Zhang, G., Liu, Y., Zhang, Y., Shang, H., Ji, H., Chen, X., Duan, Y., et al. (2015). MicroRNA-130a and -130b enhance activation of hepatic stellate cells by suppressing PPARgamma expression: a rat fibrosis model study. Biochem. Biophys. Res. Commun. *465*, 387–393.

36. Bertero, T., Lu, Y., Annis, S., Hale, A., Bhat, B., Saggar, R., Wallace, W.D., Ross, D.J., Vargas, S.O., Graham, B.B., et al. (2014). Systems-level regulation of microRNA networks by miR-130/301 promotes pulmonary hypertension. J. Clin. Invest. *124*, 3514–3528.

37. Bertero, T., Cottrill, K.A., Lu, Y., Haeger, C.M., Dieffenbach, P., Annis, S., Hale, A., Bhat, B., Kaimal, V., Zhang, Y.Y., et al. (2015). Matrix remodeling promotes pulmonary hypertension through feedback mechanoactivation of the YAP/TAZ-miR-130/301 circuit. Cell Rep. *13*, 1016–1032.

38. Xin, L., Gao, J., Wang, D., Lin, J.H., Liao, Z., Ji, J.T., Du, T.T., Jiang, F., Hu, L.H., and Li, Z.S. (2017). Novel blood-based microRNA biomarker panel for early diagnosis of chronic pancreatitis. Sci. Rep. *7*, 40019.

39. Wang, X., Li, W., Chen, J., Zhao, S., Qiu, S., Yin, H., Carvalho, V., Zhou, Y., Shi, R., Hu, J., et al. (2018). A transcriptional sequencing analysis of islet stellate cell and pancreatic stellate cell. J. Diabetes Res. *2018*, 7361684.

40. Love, M.I., Huber, W., and Anders, S. (2014). Moderated estimation of fold change and dispersion for RNA-seq data with DESeq2. Genome Biol. *15*, 550.

41. Yazdani, N., Parker, C.C., Shen, Y., Reed, E.R., Guido, M.A., Kole, L.A., Kirkpatrick, S.L., Lim, J.E., Sokoloff, G., Cheng, R., et al. (2015). Hnrnph1 is a quantitative trait gene for methamphetamine sensitivity. PLoS Genet. *11*, e1005713.

42. Li, J.H., Liu, S., Zhou, H., Qu, L.H., and Yang, J.H. (2014). starBase v2.0: decoding miRNA-ceRNA, miRNA-ncRNA and protein-RNA interaction networks from large-scale CLIP-Seq data. Nucleic Acids Res. *42*, D92–D97.

43. Nagy, A., Munkacsy, G., and Gyorffy, B. (2021). Pancancer survival analysis of cancer hallmark genes. Sci. Rep. *11*, 6047.

OMTN, Volume *27*

Supplemental information

Inflammatory-miR-301a circuitry drives mTOR and Stat3-dependent PSC activation in chronic pancreatitis and PanIN

Fugui Li, Miaomiao Wang, Xun Li, Yihao Long, Kaizhao Chen, Xinjie Wang, Mingtian Zhong, Weimin Cheng, Xuemei Tian, Ping Wang, Mingfang Ji, and Xiaodong Ma

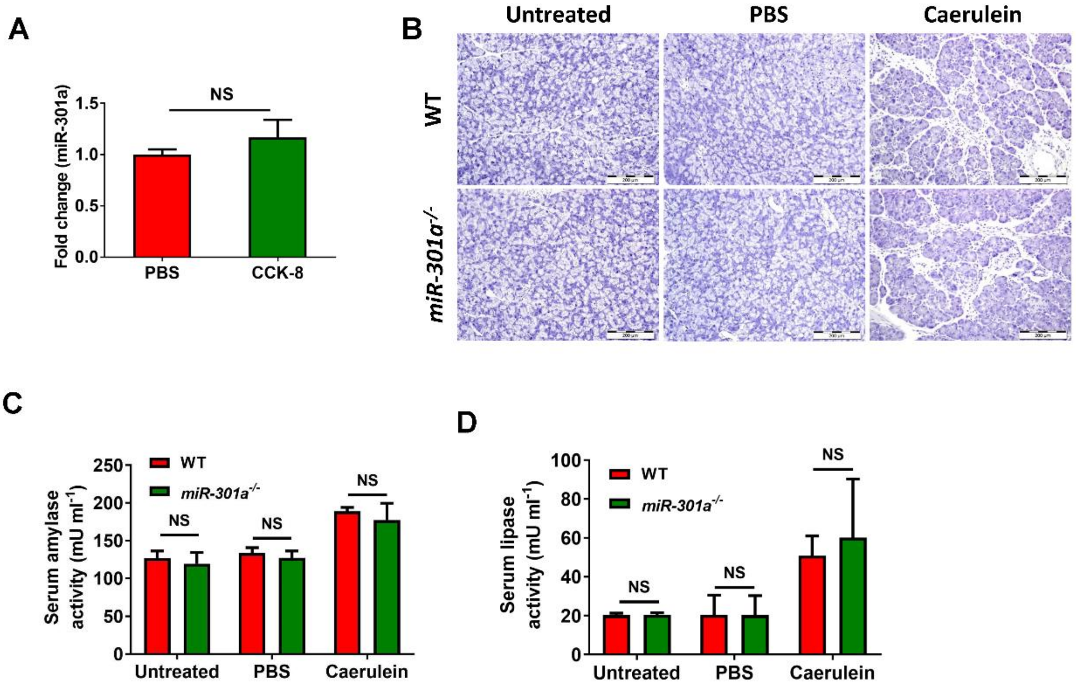

Figure S1. Response of WT and *miR-301a$^{-/-}$* mice to caerulein-induced acute pancreatitis.

(A) Expression of miR-301a in acinar cells treated with supramaximal CCK-8. Acinar cells were isolated from WT and *miR-301a$^{-/-}$* mice, and incubated for 8h with CCK-8. Total RNA was extracted from acinar cells and miR-301a expression was quantified by qPCR. **(B)** Representative histological sections of mouse pancreata stained with haematoxylin from caerulein-treated WT and *miR-301a$^{-/-}$* mice (n=6 per group). Scale bars, 200μm. **(C)** Serum amylase activity in WT and *miR-301a$^{-/-}$* mice (n=6 per group) 6h after the last caerulein injection. **(D)** Serum lipase activity in WT and *miR-301a$^{-/-}$* mice (n=6 per group) 6h after the last caerulein injection. Values are mean±s.d. NS means no significant difference between the indicated groups.

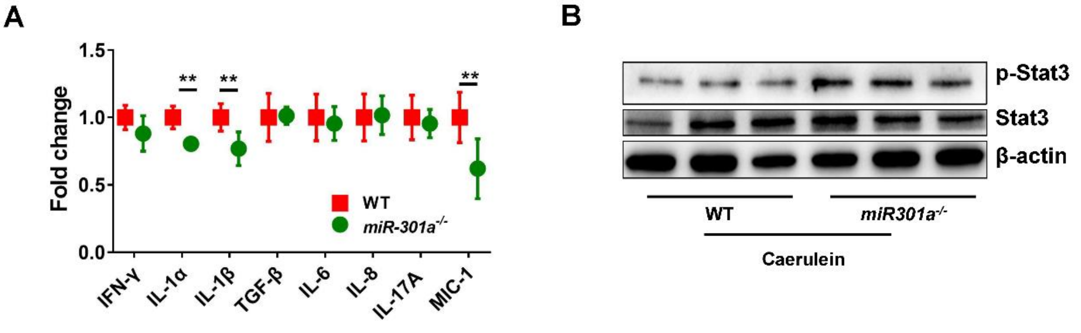

Figure S2. Stat3 activation and cytokines expression in pancreata from *miR-301a*$^{-/-}$ mice compared to WT mice with chronic pancreatitis.

(A) Cytokine gene expression in pancreatic tissues of WT and *miR-301a*$^{-/-}$ mice as determined by qPCR (n=3 per group). **(B)** Western blot analysis of pancreatic tissue lysates for Stat3 activation (n=3 per group). ***P*<0.01 indicates a significant difference between the indicated groups.

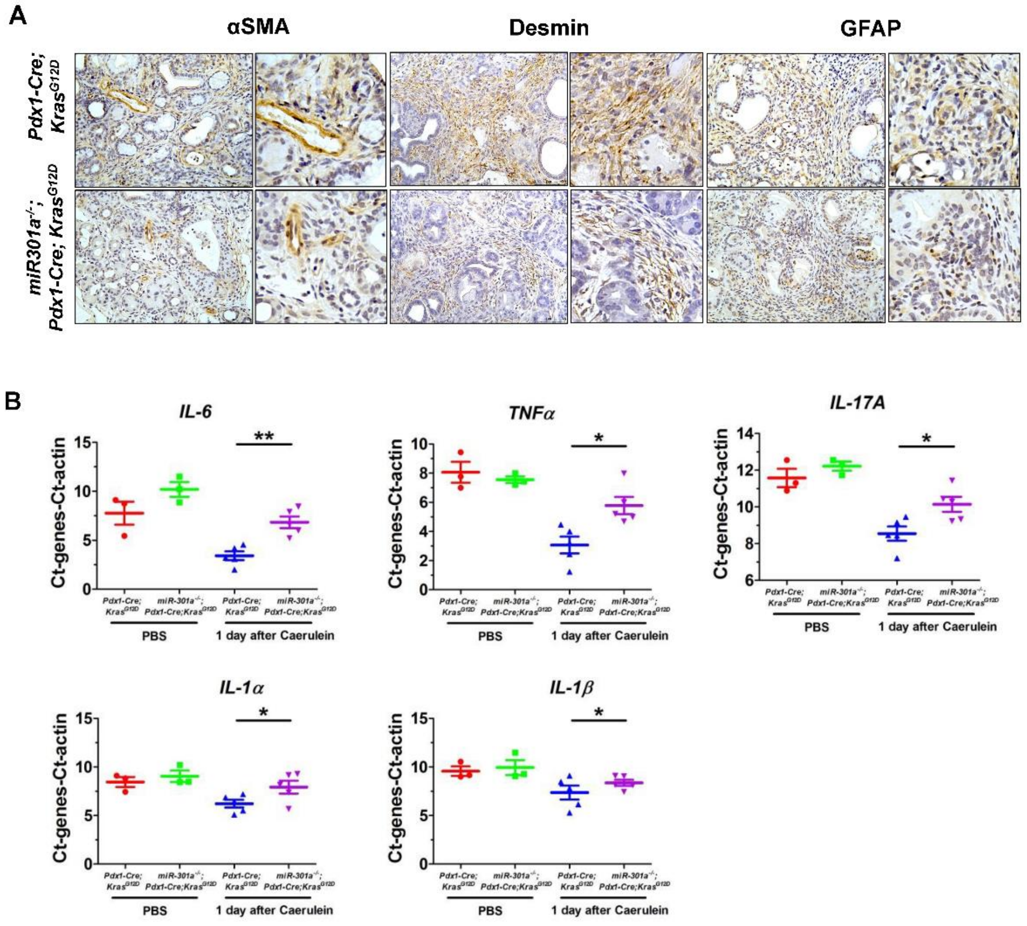

Figure S3. Characteristic markers and cytokines expression in PanIN.

(A) Immunohistochemical staining of αSMA**,** Desmin and GFAP showed activation of PSCs and increased fibrogenesis pancreata from *Pdx1-Cre;Kras*^{G12D} (n=8) and *miR-301a*$^{-/-}$*;Pdx1-Cre;Kras*^{G12D} (n=8) littermate mice at 18 weeks of age. Scale bars, 75μm. Values are means ± s.d. **(B)** Cytokine gene expression in pancreatic tissues from 9-week-old *Pdx1-Cre;Kras*^{G12D} and *miR-301a*$^{-/-}$*;Pdx1-Cre;Kras*^{G12D} mice 7 days after PBS (n=3) or caerulein treatment (n=5). ***P*<0.01 or **P*<0.05 indicates a significant difference between the indicated groups.

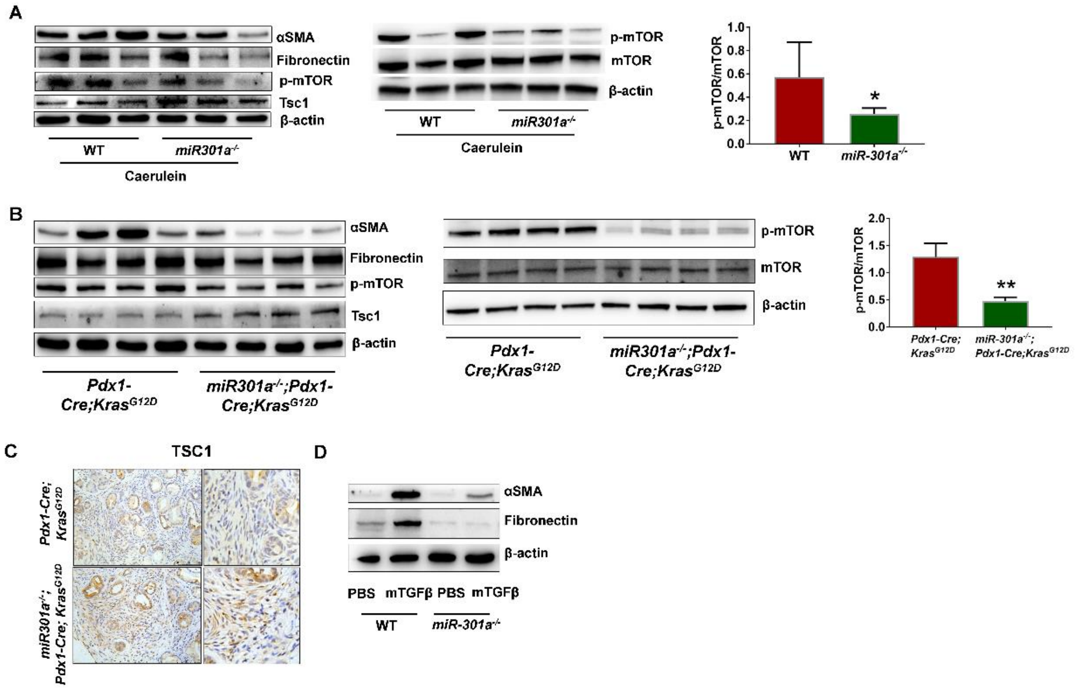

Figure S4. miR-301a/Tsc1 in chronic pancreatitis and spontaneous PanIN.

(A) Western blot shows the expression of αSMA, Fibronectin, p-mTOR, mTOR and Tsc1 in pancreatic tissues from caerulein-treated WT (n=3) and *miR-301a*$^{-/-}$ (n=3) mice. **(B)** Western blot shows the expression of αSMA, Fibronectin, p-mTOR, mTOR and Tsc1 in pancreatic tissues from *Pdx1-Cre;Kras*^{G12D} (n=4) and *miR-301a*$^{-/-}$*;Pdx1-Cre;Kras*^{G12D} (n=4) littermate mice at 18 weeks of age. **(C)** Immunohistochemical staining of Tsc1 in pancreata from 9-week-old *Pdx1-Cre;Kras*^{G12D} (n=8) and *miR-301a*$^{-/-}$*;Pdx1-Cre;Kras*^{G12D} (n=8) mice 7 days after caerulein treatment. The scale bars represent 75μm. **(D)** MEFs isolated from WT and *miR-301a*$^{-/-}$ mice were treated with TGF-β (10 ng/mL) for 24h. The expression of αSMA and fibronectin were measured by western blot analysis. ***P*<0.01 or **P*<0.05indicate a significant difference between normal tissue and tumor tissue.

A

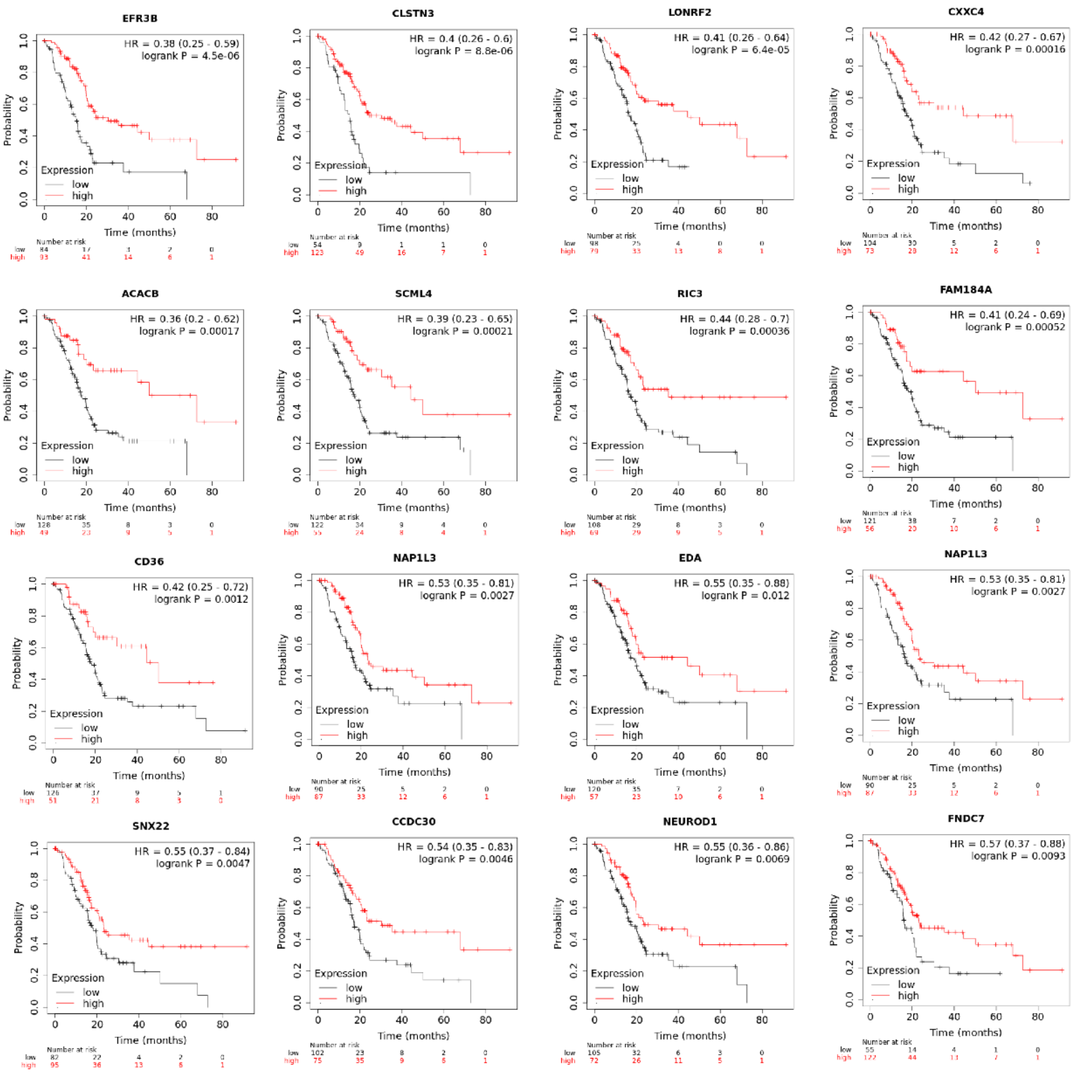

Figure S5. **(A)** Survival plots of human pancreatic patients (TCGA, PAAD) for 16 genes.

A

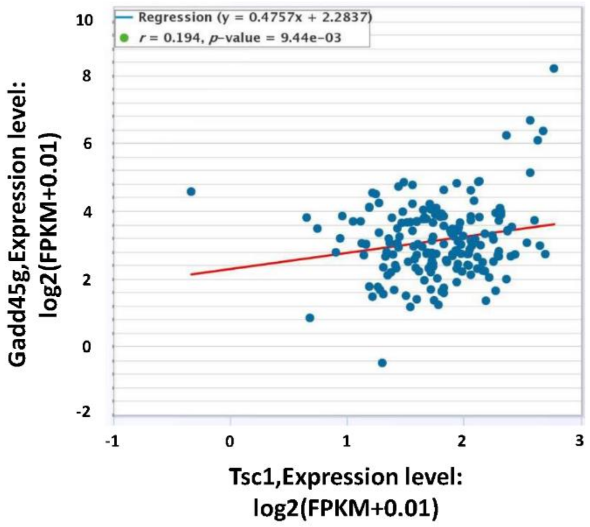

Figure S6. (A) Correlation analyses between Gadd45g and Tsc1 in pancreatic cancer patients in the TCGA database.

Table S1 The primers used for qPCR assay.

Genes	Forward	Reverse
NeuroD1	5'-AAGCCACGGATCAATCTTCTC-3'	5'-CGTGAAAGATGGCATTAAGCTG-3'
Acacb	5'-GGTAGTGGCTTTGAAGGAACTGTC-3'	5'-GATATCGTTGTTCTGGAAGCTCTCG-3'
Chst2	5'-AAGCCTACAGGTGGTGCGAA-3'	5'-CAGGACTGTTAACCCGCTCA-3'
Cd36	5'-GAGCCATCTTTGAGCCTTCA-3'	5'-TCAGATCCGAACACAGCGTA-3'
Ric3	5'-GGAAACGCCCACAGGAAGATT-3'	5'-ACCATTAGGTCCAACTCTGTTGA-3'
Ccdc30	5'-AAGGCTTGTTCAGACTAGCTCT-3'	5'-TGTAGGCTGCTGTTTGGATCT-3'
Fam184a	5'-AGCTGGCAACATCACTATTACG-3'	5'-GCTGGGCGATCTTCTTGCT-3'
Fndc7	5'-ATCACCGTAGAATGGACTACCG-3'	5'-CCTGGGGAATTAGCCACTGT-3'
Clstn3	5'-GGACAAGGCAACGGGTGAA-3'	5'-GCCACAGTCATAAGCCTGAATG-3'
Nap1l3	5'-GGGGATGAATCTGACAGCAATAG-3'	5'-TTCGGTTCAAATGACTGCCAT-3'
Snx22	5'-TACACAAGCGGATCAAGAAACG-3'	5'-TGGATGTAGGTCTCTAATCCCTG-3'
Scml4	5'-GTGCAGATGGCCGTCCATAA-3'	5'-AGCACTGGAGACTTGATCTTGT-3'
Gadd45g	5'-GGGAAAGCACTGCACGAACT-3'	5'-AGCACGCAAAAGGTCACATTG-3'
Lonrf2	5'-CAACTCGGCGAAGAAAGAAACA-3'	5'-AGGATACTGGGGATTCGTCTG-3'
Cxxc4	5'-CTGCCCGCAGAATCATTCCT-3'	5'-CAGACGCCACAGTTGATGAG-3'
Efr3b	5'-CGCCTGAAAAACTGGATCGAA-3'	5'-GGCAATACACACGTACCCATATC-3'
Eda	5'-GTGGACGGCACCTACTTCATC-3'	5’-CACCATCTTCACGGCGATTT-3'
IFN-γ	5'-ATGAACGCTACACACTGCATC-3'	5'-CCATCCTTTTGCCAGTTCCTC-3'
IL-1α	5'-CTCTAGAGCACCATGCTACAGAC-3'	5'-TGGAATCCAGGGGAAACACTG-3'
IL-1β	5'-CTCCATGAGCTTTGTACAAGG-3'	5'-TGCTGATGTACCAGTTGGGG-3'
Il-6	5'-CCTCTGGTCTTCTGGAGTACC-3'	5'-ACTCCTTCTGTGACTCCAGC-3'
TGF-β	5'-GCTACCATGCCAACTTCTGT-3'	5'-CGTAGTAGACGATGGGCAGT-3'
IL-17A	5'-TCCCCTCTGTGATCTGGGAAG-3'	5'-AGCATCTTCTCGACCCTGAA-3'
MIC-1	5'-CTGGCAATGCCTGAACAACG-3'	5'-GGTCGGGACTTGGTTCTGAG-3'
IL-8	5'-CAGCTGCCTTAACCCCATCA-3'	5'-CTTGAGAAGTCCATGGCGAAA-3'
